# Temperature-Specific and Sex-Specific Fitness Effects of Sympatric Mitochondrial and Mito-Nuclear Variation in *Drosophila obscura*

**DOI:** 10.3390/insects13020139

**Published:** 2022-01-28

**Authors:** Pavle Erić, Aleksandra Patenković, Katarina Erić, Marija Tanasković, Slobodan Davidović, Mina Rakić, Marija Savić Veselinović, Marina Stamenković-Radak, Mihailo Jelić

**Affiliations:** 1 Department of Genetics of Populations and Ecogenotoxicology, Institute for Biological Research “Siniša Stanković”–National Institute of the Republic of Serbia, University of Belgrade, Bulevar Despota Stefana 142, 11060 Belgrade, Serbia; aleksandra@ibiss.bg.ac.rs (A.P.); katarina.eric@ibiss.bg.ac.rs (K.E.); marija.tanaskovic@ibiss.bg.ac.rs (M.T.); slobodan.davidovic@ibiss.bg.ac.rs (S.D.); rakic.mina28@gmail.com (M.R.); 2 Faculty of Biology, University of Belgrade, Studentski trg 16, 11000 Belgrade, Serbia; marijas@bio.bg.ac.rs (M.S.V.); marina@bio.bg.ac.rs (M.S.-R.); mihailoj@bio.bg.ac.rs (M.J.)

**Keywords:** *D. obscura*, *Cyt b* gene, desiccation resistance, developmental time, viability, sex-ratio, mtDNA, intra-population variation

## Abstract

**Simple Summary:**

Does variation in the mitochondrial DNA sequence influence the survival and reproduction of an individual? What is the purpose of genetic variation of the mitochondrial DNA between individuals from the same population? As a simple laboratory model, *Drosophila* species can give us the answer to this question. Creating experimental lines with different combinations of mitochondrial and nuclear genomic DNA and testing how successful these lines were in surviving in different experimental set-ups enables us to deduce the effect that both genomes have on fitness. This study on *D. obscura* experimentally validates theoretical models that explain the persistence of mitochondrial DNA variation within populations. Our results shed light on the various mechanisms that maintain this type of variation. Finally, by conducting the experiments on two experimental temperatures, we have shown that environmental variations can support mitochondrial DNA variation within populations.

**Abstract:**

The adaptive significance of sympatric mitochondrial (mtDNA) variation and the role of selective mechanisms that maintain it are debated to this day. Isofemale lines of *Drosophila obscura* collected from four populations were backcrossed within populations to construct experimental lines, with all combinations of mtDNA *Cyt b* haplotypes and nuclear genetic backgrounds (nuDNA). Individuals of both sexes from these lines were then subjected to four fitness assays (desiccation resistance, developmental time, egg-to-adult viability and sex ratio) on two experimental temperatures to examine the role of temperature fluctuations and sex-specific selection, as well as the part that interactions between the two genomes play in shaping mtDNA variation. The results varied across populations and fitness components. In the majority of comparisons, they show that sympatric mitochondrial variants affect fitness. However, their effect should be examined in light of interactions with nuDNA, as mito-nuclear genotype was even more influential on fitness across all components. We found both sex-specific and temperature-specific differences in mitochondrial and mito-nuclear genotype ranks in all fitness components. The effect of temperature-specific selection was found to be more prominent, especially in desiccation resistance. From the results of different components tested, we can also infer that temperature-specific mito-nuclear interactions rather than sex-specific selection on mito-nuclear genotypes have a more substantial role in preserving mtDNA variation in this model species.

## 1. Introduction

The acquisition of a primordial free-living prokaryote ancestor of mitochondria by early eukaryotes is probably the most important step in the evolution of complex life [1]. The mitochondrial genome codes for only a few genes, but they are immensely important for the metabolism and the high energy efficiency of the eukaryotic cells. Products of the genes encoded by the mitochondrial genome act in conjunction with products of the nuclear genome. These complex interactions include cellular respiration as well as mtDNA replication, transcription and translation, all remarkably important biological processes [2].

The mitochondrial electron transport chain (METC), which is the site of the oxidative phosphorylation pathway (OXPHOS), is tightly orchestrated by the epistasis of (the genes encoded over) the two genomes. Adenosine triphosphate (ATP) is a cell fuel that is produced in the OXPHOS, by five multi-subunit enzyme complexes, four of which are comprised by the subunits encoded by both genomes [3]. METC function is dependent on the synchronised interaction between mtDNA-encoded proteins (and RNAs) and nuclear-encoded proteins that are imported in the mitochondria. These protein subunits originating from two different genomes require high compatibility analogous to a ‘lock and key’ principle to preserve their configuration and enzymatic activity. Any incompatibilities can compromise their structural and biochemical properties, which in turn can cause electron leakage and consequently oxidative stress.

Apart from the aforementioned direct influence on the OXPHOS, mito-nuclear interactions have an indirect impact on it by being enrolled in the processes of transcription and translation of mitochondrial subunits involved in METC, as well as replication of the mtDNA. Transcription of these mtDNA genes involved in the OXPHOS pathway is completely regulated by the nucleus. All polypeptides involved in the process are nuclear-coded and imported to the mitochondria. This process usually consists of two transcription factors and a single subunit mitochondrial RNA polymerase (POLRMT) which is liable for promoter-binding specificity and strength. This is the basis for transcriptional mito-nuclear interactions because the nuDNA-coded protein needs to be structurally complementary to the control region of the mitochondrial DNA, or regulation of mtDNA genes transcription is compromised [4]. Experiments have shown that when combining factors of the mitochondrial transcription machinery from different taxa, the more distant the taxa, the more deficient the transcription [5,6,7]. The authors suggest that this is due to coevolution between binding motifs in the POLRMT or splicing peptides and the mtDNA recognition sites in each species [5]. Replication of the mtDNA is also reliant on the activity of the POLRMT, as it is responsible for RNA primer creation after which replication can begin using the mtDNA polymerase complex. This way, mito-nuclear co-adaptions are accountable for mtDNA replication as well [8,9]. Apart from coding for subunits involved in OXPHOS, mtDNA also encodes for transfer RNAs (tRNAs), which cooperate with nuclear-encoded mitochondrial aminoacyl-tRNA synthetases (mt-aaRSs) in the process of translating mtDNA proteins [2,10], giving rise to another level on which mito-nuclear interactions are recognized by selection.

Since mtDNA products play such a pivotal role in the eukaryotic metabolism and normal cell functioning, scientists have long thought that any variation in the mitochondrial genome that impacts fitness would quickly be either purged or fixed by natural selection [11,12], resulting in erosion of genetic variation. This view was encouraged by the attributes of the mitochondrial genome, specifically being haploid and its exclusively maternal inheritance. A further consequence of haploidy in the absence of recombinations and heterozygosis, in which recessive alleles would be masked by dominant ones, is that all alleles are exposed to selection. Scientists thought that the high mutation rates of mtDNA are the reason for the relatively high levels of functional mtDNA variation, but theoretical models have proven that the observed levels of standing genetic variation are much higher than expected under the mutation–selection balance [13].

In the last few decades, this traditional paradigm of rejecting mitochondrial DNAs functional and evolutional relevance has shifted, with the accumulating evidence linking specific mtDNA sequence polymorphisms to substantial phenotypic effects [14]. First, signs of positive selection acting on the mtDNA in a broad spectrum of phyla have been reported by comparing the distribution of synonymous and nonsynonymous substitutions within and between species [11,15,16,17,18,19]. Second, a correlation between environmental factors and the distribution of mtDNA haplotypes has been observed [18,20,21]. Not long after, experimental evidence started to amass, pointing to fitness consequences ensued by mtDNA sequence variation [22,23,24,25].

The presence of adaptive variation in mtDNA between populations is quite easy to explain because distinct populations usually mean independent genetic pools from which the stochastic forces sieve different alleles, but also different selection pressures favouring distinct haplotypes. Even detrimental alleles can become specific for a particular population if they are compatible with arising compensatory mutations in the nuclear genome [26,27,28,29,30,31,32]. Therefore, the joint mito-nuclear interactions mentioned above are important in that respect [33].

However, the existence of stable adaptive sympatric variation is much more difficult to explain, given its haploid nature and the importance of mitochondrial genes. Over the years, many ambiguous results have been published on the subject of the role of mito-nuclear interactions in sustaining adaptive sympatric variation. While some authors [25,34] could not prove sympatric variance being maintained with mito-nuclear interactions, others proved it while using the same model species and similar experimental set-ups [26,35]. Current theoretical understanding suggests that conditions required for maintenance of adaptive within-population variance are restrictive [36,37,38]. Experimental papers on the subject have been scarce throughout the years [1,26]. A recent growing body of theoretical and experimental work has started to shed light on the subject, especially in light of cytonuclear interactions.

Negative frequency-dependent selection (NFDS) was one of the first mechanisms of balancing selection proposed to be maintaining sympatric mitochondrial variation. When it comes to this type of balancing selection, an allele’s relative fitness is inversely proportional to its frequency in the population. In a nutshell, NFDS constitutes that rarer alleles are favoured by natural selection over more common ones. The role of negative-frequency dependent selection in maintaining genetic variation in the nuclear genome is well described in the example of chromosomal inversions in *Drosophila ananassae* [39] and maintaining self-incompatibility in plants [40]. The first proposition of the NFDS’s role in maintaining mtDNA variation by acting on cytonuclear gene interactions was brought by Gregorius and Ross [38], but it was not until recently that it was hypothesised and experimentally proven that NFDS is responsible for sustaining mtDNA polymorphism in laboratory populations of seed beetle [41,42]. Furthermore, experiments on *Drosophila subobscura* are starting to give weight to NFDS effects on preserving mtDNA variation [43,44].

Sex-specific selection (SSS) is an additional balancing selection mechanism that was proposed to be maintaining standing sympatric mtDNA variation [45,46]. Theory predicts that some mito-nuclear haplotype combinations result in a higher relative fitness in one sex, while different combinations provide better fitness in the other sex. Since mitochondria are maternally transmitted, the selection can act on them only through females. Because of the different reproduction strategies and behavioural patterns and sex-specific life histories in general, males and females have different fitness, as selection only recognizes females, favouring mutations that have a positive impact on females’ fitness even though these mutations can have deleterious effects in males. This hypothesis is known as the mother’s curse [47,48]. Different mutations in mitochondria importing nuclear-encoded proteins that are favourable to males may arise, pulling corresponding mitochondria-coded proteins to compensatory adapt and adjust to the new biochemical and structural stability and to preserve the high compatibility and efficacy [49]. The idea of SSS maintaining intra-population variation was reinforced in the next couple of years by many experiments on *Drosophila* species [50,51,52], confirming that SSS acting on these mito-nuclear interactions can maintain intra-population variation.

Rand [35] developed a model of joint transmission of X chromosome and cytoplasm in *Drosophila melanogaster* and showed that maintenance of sympatric mitochondrial variation can be supported if the nuclear component of the interaction is located on the X chromosome.

Another mechanism proposed is the idea that the mitochondrial or mito-nuclear variation is shaped by differences in environmental factors that vary temporally or within habitat [22,50]. In that respect, genotype-by-environment interactions are crucial in upholding sympatric mtDNA variation. Dowling et al. [50] showed that multiple mito-nuclear haplotypes can be maintained by epistatic interactions between mitochondrial and nuclear genes in a random mating population. Their findings show that the adaptive value of specific mito-nuclear combinations are determined by the environment in which they are expressed. Furthermore, Willet et al. [53] have shown the substantial influence of temperature and light regime on selection on interpopulation mito-nuclear crosses of intertidal copepod *Tigriopus californicus*. In this study, it is advocated that differences in protein interactions at varying temperatures are the reason why specific mito-nuclear combinations are favoured at a given temperature range. Oscillating temperature settings over space and time could act to maintain not only mitochondrial but mito-nuclear variation within populations, when this mtDNA × nuDNA × environment epistasis occurs [54].

Thus, different mitochondrial polymorphisms can be auspicious in different extrinsic terms, for example, temperature, or different intrinsic conditions, be it a different nuclear genetic background (if possible, X linked) or different sex. In addition, the mere frequency of the polymorphism can make it more or less favourable.

Insects have long served as suitable models to measure the adaptive significance of mitochondrial or mito-nuclear variation. Generally, their generation time is short, making it possible to fully replace the existing nuclear background with the desired one by multi-generational backcrossing in a relatively short time [41,52,54,55]. The experimental model used in this study, *Drosophila obscura*, possesses a high level of sympatric mtDNA variation across the species range [56]. Therefore, it gives an excellent possibility to test the adaptive significance of sympatric mtDNA variation, and consequently forces that maintain it. Our preliminary work on desiccation resistance, in this model species [57], identified the importance of temperature-specific effects on mito-nuclear variation, and to a lesser extent, the effects of SSS. In the present study, we use four Experimental Blocks (EBs), each representing variation collected within a specific natural site. Each block has three distinct mtDNA haplotypes and three distinct nuclear genetic backgrounds that are combined in nine possible experimental mito-nuclear introgression lines (MNILs). These MNILs were subjected to measurements of a set of life-history traits (both larval and adult) at two different temperatures. This way, we disentangle the specific effect of mtDNA on fitness and its dependence on nuclear genetic background, experimental temperature and sex of the individual. Ultimately, we discuss the importance of mito-nuclear interactions, genotype-to-environment interactions, and sex-to-genotype interactions, and we identify balancing selection mechanisms that maintain sympatric variation in mtDNA in natural populations of this model species.

## 2. Materials and Methods

Starting material for this study consisted of isofemale lines (IFL) of *Drosophila obscura* that were constructed from females collected in the wild and that were previously genotyped for the *Cyt b* gene [56]. These IFLs were maintained in the laboratory on a standard corn-meal medium for multiple generations. All IFLs used were previously tested and were negative on *Wolbachia*. In addition, IFLs used in this experiment were jointly analysed for microbiota composition, and no maternally transmitted bacteria were found [56]. Four experimental blocks (EB) were formed, each from a specific locality. Three distinct sympatric haplotypes per block, representing IFLs from the same population were chosen in order to construct 9 MNILs.

The first EB consisted of three haplotypes O1, O2 and O8, from the ST population collection site 1 [56]. Experimental block II was formed by backcrossing haplotypes O2, O9 and O10, which originated from the ST population collection site 2. Combinations of haplotypes O2, O4 and O3 belonging to the population SS, were used to make MNILs for the EB III. The MNILs of the fourth EB were constructed using haplotypes O2, O3 and O18 which come from the SG population. Specific IFLs were renamed to avoid confusion between EBs, as given in Table 1.

**Figure 1:**
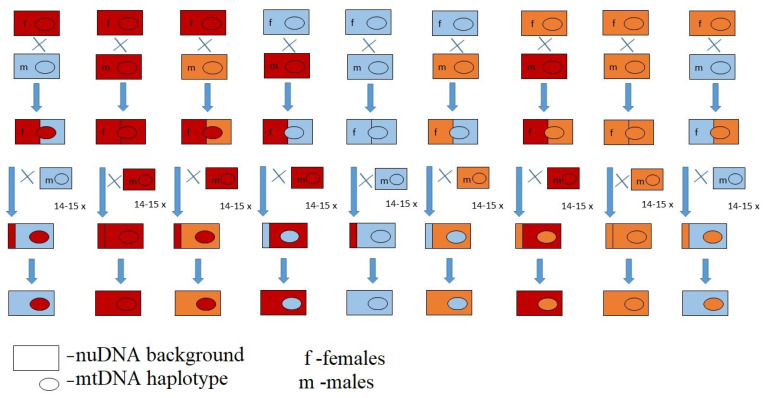
Full backcrossing design for each experimental block.

For each population, three selected IFLs were backcrossed for 14 generations, creating nine MNILs with all combinations of mitochondrial haplotypes (mtDNA) and nuclear genetic backgrounds (nuDNA). For the backcrossing procedure, we kept the vials with fly pupae in the dark such that we can collect virgin flies every morning. There is scarce information on the reproductive behaviour of *D. obscura*; thus, we tested the methodology for collecting virgin individuals used for *D. subobscura* [25,52]. From the pilot experiment, we concluded that *D. obscura* flies will not mate in the first 24 h after eclosion when being in total dark, since no flies oviposited upon being collected from a dark box every 24 h. Each MNIL was created by mating 10 virgin females of a specific haplotype with 20 virgin males with the desired nuclear genetic background. The full backcrossing design is shown in Figure 1. After 14 generations, flies were sequenced for the *Cyt b* gene again to verify that all of the final 36 MNILs possess the appropriate haplotype.

**Figure 2:**
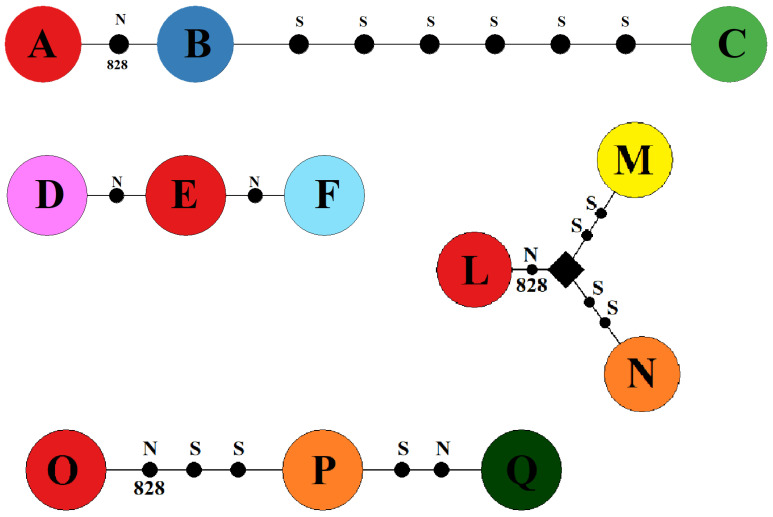
Haplotypes A, B and C from EB I; haplotypes D, E and F from EB II; L, M and N from EB III; haplotypes O, P and Q from EB IV; identical haplotypes are coloured with the same colour. S, synonymous mutation; N, nonsynonymous mutation; 828 A > G, a specific nonsynonymous substitution that separates the two groups of haplotypes.

In each block, we wanted to compare different mitochondrial haplotype triplets; in some, we compared specific single nucleotide polymorphisms that distinguish between most prevalent haplogroups, while some pairs differ in 6 or more mutations [56]. The scheme of differences between haplotypes in each of the four EBs is given in Figure 2.

In order to compare the fitness of the different combinations of mito-nuclear haplotypes, we conducted two grand experiments with several fitness components tested, in different generations due to a large number of individual flies of both sexes needed for each experiment. All experiments were conducted at two different temperatures. In all experiments for every block, we modelled three comparisons of haplotype pairs as well as the whole block with three different haplotype pairs compared together.

### 2.1. Desiccation Resistance

Desiccation resistance was the first conducted experiment after the 14th generation of backcrossing, two experimental temperatures were 16 and 19 °C, while the air humidity was set at 30%. For every specific MNIL, we had up to 40 flies per sex, for each of the two experimental temperatures. In some MNILs, we could not collect 40 flies per sex of the desired age. We had 38.8 females and 37.6 males on average for each MNIL per temperature. Individual virgin flies 5 to 7 days old were placed in small plastic modular containers with small holes for air circulation, where each module is being capped with the next making a total of two columns with 20 connected plastic containers per sex for each MNIL, for easier inspection. Containers were placed in two different rooms with regulated temperature and air-humidity levels. After the experiment was set, the flies were inspected every hour until effectively all flies had died. Mortality was determined by the inability of the *Drosophila* to keep an upright position or stand up after the plastic container had been shaken. In 4 EBs, each containing 9 MNILs, two temperatures, two sexes and up to 40 individuals had around 5500 flies tested for desiccation resistance.

### 2.2. Egg-to-Pupa-to-Adult Viability, Developmental Time and Sex Ratio Experiment

This experiment was conducted after the 15th generation of backcrossing, on two developmental temperatures (15 °C and 20 °C). For each MNIL, 40 virgin females and 60 virgin males 6–7 days old were placed in a bottle with a standard corn-meal medium for 5 days to mate. Another bottle with the same number of flies was kept as a backup. After 5 days flies were relocated to the new empty bottle which had a Petri dish instead of the cap. New bottles were placed upside down, standing on the Petri dish with corn-meal medium and yeast paste for flies to oviposit eggs. Each morning during the set-up phase, we replaced the old Petri dishes with the new ones. From the Petri dishes, we collected the oviposited eggs under binoculars and placed 20 eggs per vial for the viability experiment. For every MNIL, we made 30 replicas containing 20 eggs for each of the two experimental temperatures. Vials containing eggs were kept at two thermo-regulated chambers under constant temperature, light and dark cycles (15 °C and 12 h light/12 h dark regime and 20 °C and 16 h light/8 h dark regime) to imitate different seasons. Every two days we shuffled the vials inside the chambers to make sure that the temperature is constant for each vial. To sum up, with 4 EBs, each containing 9 MNILs, two temperatures, and 30 replicated vials with 20 transferred eggs we had over 43,000 eggs (4 × 9 × 2 × 30 × 20 = 43,200).

To measure developmental time 10 vials out of the 30 replicas for each temperature for each of the 36 MNIL, were randomly chosen, to be checked every day for hatched flies. Emerging adult flies were counted and sexed every day (in an air-conditioned room of the desired temperature) until the end of the experiment, with the number of pupae inside each vial counted as well. For the rest of the replicas, the number of adult flies, males, females and pupa was counted at the end of the eclosion after day 36.

### 2.3. Statistical Analysis

All statistical analyses were conducted using R v.4.0.3 [58]. All Figures were made in R using the ggplot2 package [59].

Desiccation data were analysed using a Cox proportional hazards model [60] using survival package v.3.2-13 [61] for each EB individually as well as for each of the three pairwise comparisons per EB, with no censoring since all flies died in the experiment. Mitochondrial haplotype (mtDNA), nuclear background (nuDNA), sex and temperature were used as fixed effects with all interactions of the four factors used as well (mtDNA × nuDNA × sex × temp). The proportional hazards assumption was checked with the cox.zph function, and where violated, the corresponding factors were stratified. Cox proportional models for EBs I and III were already presented at the 1^st^ international electronic conference on Entomology [57]. Here, we present extended models with all factors and interactions included, in order to make the results of all four blocks comparable.

Developmental time data were analysed with a general linear model using the lmer function from the lme4 package in R [62]. Full model with REML estimation and type III sums-of-squares was fitted with all interactions of fixed effects factors (mtDNA, nuDNA, sex and temperature) and replica number as random effect factor for each EB as well as for each pairwise comparison inside the EBs. LmerTest package [63] was used to obtain *p* values in ANOVA model fits.

Viability per vial was analysed with generalised linear models using the glm function in R. Egg to pupa (EtP), pupa to adult (PtA) and egg to adult (EtA) viability was scored for each pair of haplotypes compared respectively and for all four blocks apiece. Models had mitochondrial haplotype, nuclear background and temperature as fixed effects and all interactions of the three factors. All three component models, (EtA, EtP and PtA) had binomial error distribution and used the number of eggs/pupae per vial as the denominator.

Sex ratio data calculated as a proportion of males was analysed using the same general linear model with binomial error with the total number of eclosed adults per vial as the denominator. All pairwise comparisons were modelled individually, but also pulled together within each EB.

## 3. Results

### 3.1. Desiccation Resistance

Mean desiccation resistance times for four EBs are given in Supplementary Figures S1–S4. When modelling desiccation resistance, we found sex to have a significant effect on survival times as we expected. This is because female *D. obscura* flies are generally bigger than their counterpart males. Greater surface area to volume ratio means increased exposure to the environment, which makes survival in dry conditions harder. Flies of both sexes survived longer on the lower temperature in all four EBs. In all EBs, the differences between the groups are much more pronounced at 16 °C.

Here, we jointly analysed Cox proportional model data on desiccation resistance from four EBs, two of which (I and III) were presented at the First International Electronic Conference on Entomology [57] (https://sciforum.net/paper/view/10522, accessed on 22 December 2021; I = A, II = B, III = C, IV = L, V = M, VI = N). The ANOVA of Cox proportional hazards models for each EB, both pairwise comparisons and the whole block are given in Table 2, Table 3, Table 4 and Table 5. Across all blocks, there were some comparisons that had their proportional hazards assumption violated. All factors whose hazard functions were not proportional to other factors in the comparison were stratified accordingly. In the first EB, mitochondrial haplotype had a significant effect on desiccation resistance in two out of three comparisons [57], as well as in the second EB. In the third [57] and fourth EB, mtDNA was significant in only one out of three haplotype comparisons. In total, mtDNA significantly influenced desiccation resistance in six out of twelve comparisons. The nuclear background was also highly significant in five out of the seven un-stratified comparisons. In the first EB, sex was highly significant in all comparisons, while in the latter three blocks, it was significant in only one comparison per block (In total: 6/11). Furthermore, the combination of sex and mtDNA also influenced survival in the desiccation experiment, as it was significant in two out of three comparisons in the first three EBs and one out of three pairs in the fourth EB. Temperature, as was expected, had the most substantial effect on desiccation survival time, as it was highly significant in all comparisons in which its proportional hazard assumption was not violated (7/7 comparisons). The interaction term of temperature and mitochondrial haplotype had an impact on desiccation resistance, being significant in all three pairwise comparisons in EBs I and III [57], while in the II and IV EB, it was statistically significant in two-thirds and one-third of comparisons, respectively. Mito-nuclear haplotype combination mtDNA × nuDNA had an even bigger effect on the desiccation resistance than the mitochondrial haplotype. In EBs I and III, it was highly significant in all three comparisons [57], while in EBs II and IV comparisons it was significant in one comparison respectively. Sex × mtDNA × nuDNA interaction did not prove to be influential for survival under desiccation stress, as in the first two EBs no comparisons showed significance, and only one out of three comparisons in EBs III and IV. Mito-nuclear haplotype and temperature interaction, conversely, influenced the flies survival time significantly in eight out of twelve total pairwise comparisons with all of the comparisons in the third EB being highly significantly influenced by it. The combination of mitochondrial haplotype, sex and temperature produced different results in different EBs, as it showed no connection to survival time in the first two EBs, while it showed significant influence on survival time under desiccation stress in two out of three comparisons in each of the last two EBs. The highest interaction term with all four factors (mtDNA × nuDNA × sex × temp) had a mild effect on survival time as it was significant in one out of three comparisons in the EBs I, II and III and two out of three pairs in EB IV.

When analysing EBs as a whole (comparison of all three haplotypes per EB), nuDNA and temperature as individual factors were significant in all blocks in which they were not stratified. Sex was significant in two out of three blocks. Mitochondrial haplotype was significant in two out of four blocks, while the combination of mtDNA and nuDNA was highly significant in all four EBs. Interaction terms of mtDNA × sex, as well as mtDNA × temperature, were highly influential on desiccation resistance in three of four EBs each. Conversely, mito-nuclear interaction with sex was significant only in the fourth EB. The combination of mito-nuclear haplotype and temperature was clearly associated with desiccation stress survival time in all EBs. Genotype × sex × temperature, both only mitochondrial, and the combination of mtDNA and nuDNA were statistically significant in three EBs each.

### 3.2. Developmental Time

Mean developmental times for all four EBs are presented in Supplementary Figures S5–S8. Overall, males and females had similar developmental times across all EBs, with some genotype combinations being favoured in males and others in females. Temperature, conversely, had the most substantial influence on the developmental times across all EBs, with all groups developing notably faster on the higher temperature, as was expected.

ANOVA results of GLM of developmental time for four EBs are given in Table 6, Table 7, Table 8 and Table 9. Temperature was significant in all comparisons across all EBs. Mitochondrial haplotype, as well as nuclear background and sex, showed different impacts on the developmental time in different EBs. In the first EB, mtDNA was significant in one comparison, while nuDNA and sex were significant in two. Both mtDNA and nuDNA were highly significant in all three comparisons in EB II, while sex was in only one. Sex showed no significant influence on developmental time in the third EB, while both nuDNA and mtDNA were significant in one out of three comparisons. EB IV had two-thirds of comparisons statistically significant for mtDNA and one-third for nuDNA, while sex was highly significant in all three pairwise comparisons. Mito-nuclear interaction affected the developmental time in two comparisons in EBs I and IV and one comparison per block in II and III. Sex × mtDNA interaction was not as influential as sex and mtDNA are individually, as it had an effect in only one pairwise comparison in EBs II and IV. Moreover, sex × mito-nuclear interaction was also not important for the developmental time as it showed no effect in three EBs, while in the third EB, it was significant in two comparisons. Genotype × temperature interaction (mtDNA × temp and mtDNA × nuDNA × temp) is not pivotal for the developmental time as they were both statistically significant in only three comparisons out of the total twelve, across all EBs. Sex × mtDNA × temperature was also proven to be noncrucial for development time as it showed statistical significance in only one comparison in the second EB. In addition to that, the highest interaction term with all four factors included showed significant influence on developmental time in only two pairwise comparisons out of the total twelve.

When modelling EBs as a whole (comparing all three haplotypes per EB), temperature and nuclear background had the biggest impact on developmental time, being highly significant in all four blocks. Sex was significant in three EBs, while mtDNA and mtDNA× nuDNA interactions with sex showed no connection to the developmental time in none of the four blocks modelled. The combination of mtDNA, temperature and sex was also nonsignificant in all four blocks. While mtDNA was a significant factor in two EBs, mtDNA × nuDNA interaction was significant in three. Interaction terms containing temperature and genotype (mtDNA and mtDNA × nuDNA) showed a statistically significant influence in two and one EBs respectively. The highest interaction term with all four factors was significantly influencing developmental time in half of the four EBs modelled.

### 3.3. Viability

#### Egg-to-Adult

Mean egg-to-adult (EtA) viability scores for the four EBs are presented in Supplementary Figures S9–S12. In the viability experiment, we noted that mito-nuclear combinations that consisted of an mtDNA haplotype on its own nuclear background (e.g., DD, LL, QQ), usually scored the lowest viability, except in the first EB where they were usually most viable.

The results of ANOVA on EtA viability from all pairwise and whole block comparisons are presented in Table 10. The nuclear genetic background had the most substantial effect on the EtA viability as it was highly significant in all pairwise comparisons across all EBs. Temperature as well was crucial for EtA viability, being statistically significant in a total of nine comparisons across all EBs. The effect of mtDNA on viability was more variable, conversely. In the first EB, it showed a significant effect in only one of three comparisons, while in the II and III, it was significant in two of the pairs. In the fourth EB, mtDNA was significant in all pairwise comparisons. We found that mito-nuclear interaction showed substantial influence on EtA viability as it was statistically significant in nine out of the total twelve comparisons across all EBs. Different mtDNA haplotypes on different temperatures had significantly different EtA viability in eight out of twelve comparisons, making this interaction also important. Mito-nuclear genotype × temperature interaction had different results in different EBs. In EB II its effect was significant in all comparisons, while in the IV EB, it was significant in none. EBs I and III had one and two out of three comparisons statistically significant respectively.

When modelling EBs as a whole (comparing all three haplotypes per EB), mtDNA was significant in all four EBs, furthermore, nuDNA, temperature as factors as well as their interaction terms with mtDNA showed statistical significance in all four EBs. The three-factor interaction term was also highly significant in all EBs.

The model for the egg-to-pupa (EtP) viability had similar results as the EtA model with all factors discussed being highly statistically significant. Pupa-to-adult (PtA) viability was high, with most of the individuals that reached the pupal stage reaching adulthood. Results for EtP and PtA viability are given in Supplementary Tables S1 and S2.

### 3.4. Proportion of Males

The mean percentage of males for all genotype combinations inside all four EBs on two temperatures are given in Supplementary Figures S13–S16. A skewed proportion of males towards females was observed in a few experimental MNILs. This effect was most pronounced in MNILs with M nuclear genetic background with almost all combinations of mito-nuclear haplotypes on both experimental temperatures having as little as 5% of hatched individuals male. The only exception is the MM combination at 15 °C where the percentage of males spikes up to around 12%, which is still considered low (Supplementary Figure S15). In the fourth EB, distortion in the sex ratio was also detected, although not as pronounced and not as obvious as in the EB III, as there were significant differences between the same MNILs on two experimental temperatures (Supplementary Figure S16). In only one case did the proportion of males decrease to as low as 12% in the QO MNIL at 20 °C, while that same MNIL on the lower temperature had a proportion of males around 40%. This effect was most noticeable on the O nuclear background, but also in the OQ MNIL which has Q nuclear background and O mtDNA haplotype.

The results of ANOVA on the percentage of males from all pairwise and whole block comparisons are presented in Table 11. The results on percentage of males indirectly reflect influence of sex interacting with other factors on egg-to-adult viability. None of the factors modelled for the EB I were significant for this fitness component, both in pairwise comparisons inside the first block and when modelling the block as a whole. Mitochondrial haplotype was significant in three out of the nine remaining pairwise comparisons in the latter three EBs. The nuclear background showed influence on the proportion of males in two out of three comparisons for each of the III, and IV EBs. The temperature conversely, showed an effect on the proportion of males in two out of three pairwise comparisons inside EBs II and III. Out of the interaction terms, mito-nuclear interaction proved to be most influential as it was significant in five out of twelve comparisons, with mtDNA × temp being significant only in two comparisons within the second and fourth EB. The three-factor interaction term was significant in three out of six pairwise comparisons within EBs III and IV and none in the first two EBs.

When analysing EBs as a whole (comparing all three haplotypes per EB), more statistical power does not result in more statistical significance as we obtain the same results. Mitochondrial haplotype was significant only in II and III EB, while the temperature was only in III. The nuclear background was still significant in EBs II, III and IV, as well as mtDNA × nuDNA. Temperature × mtDNA interaction was still only significant in IV EB, and the three-factor interaction influenced the proportion of males in III and IV EB only.

## 4. Discussion

We chose haplotypes for this experiment based on the differences in the *Cyt b* gene. One of our goals was to see if there are principal differences between the number and type (synonymous vs. nonsynonymous) of polymorphisms and the degree of fitness differences achieved by bearers of different mito-nuclear combinations.

First, we wanted to examine whether crossing lines with different *Cyt b* 828A > G variants, a nonsynonymous substitution that divides two large groups of haplotypes in *D. obscura* [56], will result in a lower relative fitness. This was completely confirmed in EB I where we compared MNIL A which has G on bp 828 to MNILs B and C which have A, additionally, B and C have six synonymous mutations between them. Practically all fitness components in this EB (except pct. of males that had no factor significant in EB I) showed that the greater the difference in mtDNA sequence the greater the significance and impact on MNILs relative fitness. Comparison with the most different haplotypes (A and C) had both the mtDNA and mtDNA × nuDNA interactions as significant in the largest number of components assayed and the most similar haplotype comparison (B and C) had those factors as significant in the fewest components tested.

Contrary to these results, in other EBs fitness differences between haplotypes with aforementioned 828A > G, and other substitutions were not as correlated with the magnitude and type of sequence variation. Although we obtained predictable results in some components tested both for mtDNA and cytonuclear interaction effects, it was nowhere near the uniformity seen in the first EB, where it was almost as a rule. For some components, synonymous mutations proved more influential on fitness than nonsynonymous ones. Moreover, in some pairwise comparisons, combinations of more distant haplotypes showed greater relative fitness than combinations of close haplotypes.

It has been known for some time that even synonymous mutations have fitness consequences, which may sometimes be greater than nonsynonymous ones [64]. It is also generally assumed that the greater the number of mutations the bigger the phenotypic differences between the MNILs, but that may not always be the case. The discrepancy in our work between different MNILs could be because the lines we used were sequenced only for the *Cyt b* gene, and all other differences between mitochondrial DNA were not known. Thus, what looks more similar or more divergent when we look only in *Cyt b* haplotypes may not be the case for the whole mitochondrial genome. In our experiment, we had the same haplotype pairwise comparison within the third and fourth EB, as LN and OP are the same combinations of *Cyt b* gene haplotypes but coming from different populations. In almost all the components tested, we had contrasting results when comparing these two sets of identical haplotype comparisons. Apart from all the differences outside the *Cyt b* gene that were not screened in our work, these MNILs, with the same *Cyt b* haplotype originate from two different populations, and in turn should have completely different nuclear genetic backgrounds. This makes their comparisons difficult and further explains the discrepancy in our results.

During the backcrossing procedure that preceded the experiments, in IFL M we noted an unusually high percentage of females, while in O IFL, the observed portion of females was slightly elevated. The effect was apparent enough that we had difficulties collecting half as many M male flies, for the crossing procedure, as only about 5% hatched individuals were male. This effect is associated with the nuclear genome since the sex ratio experiment showed that this distortion is present only in MNILs that had M nuclear background. Maternally transmitted microorganisms can be excluded as a factor that causes this distortion by male killing or feminization, since it is not associated with a particular mtDNA haplotype which is expected to be transmitted jointly with maternally transmitted microorganisms. In addition, known maternally transmitted organisms were excluded with molecular genetic techniques including microbiome sequencing [56]. Compared to other MNILs from the third EB, MNILs with M nuclear background did not show lower viability that could be caused by embryonic lethality in males. Rather, at 15 °C, they showed much higher EtA viability indicating the exclusion of Y chromosomes before fertilization.

This sex ratio distortion (SRD), in particular MNILs, which is associated with nuclear genetic background is probably caused by a meiotic drive mechanism. This sex ratio distortion is frequently found in *Drosophila* [65]. The first-ever case of the meiotic drive has been documented in *D. obscura* [66]. Gershenson found sex distortion in two out of nineteen IFLs formed by females collected in the wild. Studious experiments on deviations in the percentage of males that hatched from crossing different *D. obscura* lines led him to a conclusion that a gene localized on the X chromosome prevents the genesis of functional spermatozoa without X chromosome. The meiotic drive has been confirmed in a broad range of phyla, and papers on different *Drosophila* species showed that there are more than a few molecular mechanisms for it to cause SRD [67,68].

Although the majority of MNIL with M nuclear background had around 5% of males eclosed in viability experiment, MM MNIL, scored a twofold increase (12%) at a lower temperature. This observation is in line with the findings that sex ratio distortion is extremely temperature-sensitive, as spermatogenesis in higher temperatures results in a higher percentage of X chromosome bearing sperm [69,70]. Although the sex ratio was initially skewed in some MNILs, probably due to meiotic drive, our experimental design enabled us to capture and quantify the effects that mtDNA and nuDNA had on the survival of hatched individuals of a specific sex even in these lines with intrinsic skewed sex ratio.

This study supports a growing body of evidence of non-neutrality of mitochondrial DNA variation [26,71,72], and more importantly, our results give weight to the adaptive significance of intra-population variation in mtDNA [51,73,74]. As we hypothesized, almost all of our models for different fitness components showed that mito-nuclear interactions are more important as units for selection to act on than mitochondrial haplotypes on their own, as our results suggest. This should come as no surprise, considering that the *Cyt b* gene is part of the respiratory complex III, which includes subunits coded by both genomes. In addition, as noted previously, haplotypes probably have differences in genes that comprise the other three of four complexes that have subunits coded by both genomes. If an mtDNA haplotype is coupled with a non-matching nuDNA background, a decline in adaptive value is expected, as the subunits from two genomes have to be co-adapted for the optimal energy production in the mitochondria.

As expected, our experimental model identified sex-specific differences in the fitness of bearers of different mtDNA haplotypes. While this effect was significant in seven out of twelve pairwise comparisons in the desiccation experiment, this male-specific mutational load was noticeable in only two comparisons for the developmental time component. This effect was indirectly measured as an effect of mtDNA in the sex ratio component where it was significant in three out of twelve pairwise comparisons. This observation is in line with the mother’s curse hypothesis, a phenomenon frequently found while measuring the effects of mtDNA on life history [47,48,50,55]. Due to maternal inheritance of mtDNA, mutations that are disadvantageous only in males, and have no effect or are advantageous to females, cannot be purged by natural selection.

One of our goals was to test whether different combinations of mitochondrial and nuclear genomes show different fitness ranks depending on the sex of the individual. This finding would support the theoretical presumptions that SSS is responsible for maintaining stable sympatric mitochondrial and mito-nuclear variation [45,46]. Experimental support for this type of balancing selection comes from several previous experiments. When analysing cytonuclear interactions between the X chromosome and mitochondrial DNA, Rand [35] observed the action of SSS in *D. melanogaster*. In a viability experiment with 25 mtDNA haplotypes scored on three nuclear genetic backgrounds, Dowling et al. [50] found the interaction of mtDNA × nuDNA × sex significant, but only in the first out of three repeated EBs. In the second EB, they could not test this interaction due to missing data, but in the third EB and in overall analyses (across EBs), they did not find evidence of sex specificity of mito-nuclear effects on viability. Jelic et al. [52] analysed a series of key life-history traits of *Drosophila subobscura* MNILs made from three sympatric mtDNA haplotypes. They unequivocally found sex-specific effects (mtDNA × nuDNA × sex) in two experimental modules for adult longevity and indirectly in one module (mtDNA × nuDNA) for egg-to-adult viability when analysing the proportion of males hatching [52]. Conversely, they found no evidence of SSS in any of the experimental modules for desiccation resistance. Similarly, using seed beetles *Acanthoscelides obtectus,* Đorđević et al. performed mortality assays and tested mito-nuclear effects on survival [55]. Although they did not obtain significant mtDNA × nuDNA interaction in their models, mtDNA × nuDNA × sex interaction was significant in model for variance in lifespan, but not in two survival models. In their work, they also analysed the effects of mtDNA and nuDNA as well as sex on the activity of METC complexes. Variation in ETC activity was significantly influenced by sex-specific mito-nuclear interactions in METC complexes I and II, contrastingly they did not find this interaction significant in complexes III and IV. In our experimental design, the role of SSS on mito-nuclear variation was scored directly in desiccation resistance and developmental time as a significant effect of interaction between mtDNA × nuDNA × sex. Conversely, a significant interaction between mtDNA × nuDNA for a sex ratio of hatched adults is also an indirect measure of SSS. In our model species, we found the interaction between mito-nuclear genotype and sex to be statistically significant only in two out of twelve pairwise comparisons for developmental time and desiccation resistance experiments each (if desiccation resistance is scored jointly with previously analysed blocks [57]). Signature of SSS was observed in five out of twelve comparisons in the sex ratio experiment. Our findings support theoretical presumptions that SSS is involved in the maintenance of sympatric mtDNA variation.

The aim of this study was also to test whether different mitochondrial haplotypes or combinations of mitochondrial and nuclear genomes show different fitness ranks depending on the experimental temperature. This finding would support the idea that temperature variation may promote stable sympatric genetic variation. Temperature is of key importance in metabolic processes. Numerous papers on the subject point to the particular sensitivity of OXYPHOS enzyme complexes to temperature [54,75,76,77]. The connection between mtDNA variation and the temperature has been observed as a clinal shift of haplotype frequencies along latitude [78,79,80] and altitude [81,82]. Additionally, the optimal function of subunits coded by two different genomes may depend on the thermal environment that the reactions are taking place. While some combinations of mtDNA and nuDNA may be supreme in one thermal environment that may not be the case in others.

Using seed beetles Callosobruchus maculatus, Immonen et al. [54] had different mtDNA haplotypes compete on two different experimental temperatures. Their results claim that temperature is influencing mtDNA evolution to some extent, most likely through mito-nuclear interactions. Similarly, another study [22] measuring EtA development time on the same model organism and two temperatures showed the significance of G × G × E interactions. This effect of temperature-specific fitness of MNILs was found once more, [41] using the same experimental lines in another experiment, measuring metabolic rate. Similarly, Rand [83] using Drosophila as a model found that altered dietary or oxygen environments modify the fitness of mito-nuclear haplotypes.

Research that analyses the fitness of sympatric mitochondrial variation in regard to the extrinsic environment including temperature is scarce [14]. For example, Dowling et al. [50] analysed a single panmictic laboratory population and showed that multiple mitochondrial haplotypes can be preserved within it. Their experiment consisted of three repeated measures (three blocks) of the same experiment, and as they suggest relative fitness of the cytonuclear combination is dependent on the environment that they exist in, as they find the effect of the block to be substantial. They attributed this to the unforeseeable heterogeneity of environmental factors across blocks [50]. The effects of temperature or other environmental factors on fitness ranks of mito-nuclear genotypes between populations suggest [14,22,50] that this mechanism could potentially support sympatric variation as well. Although the haplotypes used by Immonen et al. [54] originate from geographically distant populations, their haplogroups have all been found to segregate sympatrically in the same West African population, which gives more support to the above claims of temperature-specific epistasis.

Data for our model species included temperature as an environmental factor in the experiments, and we showed that the genotype × temperature interaction has a significant effect on *Drosophila* fitness in all components assayed in this work. This effect was especially important in desiccation resistance (if analysed jointly with [57]), and viability experiments, with both mtDNA × nuDNA × temp and mtDNA × temp interactions being highly significant in both pairwise and whole block models. The abundance of this type of interaction in our data supports the presumption based on interpopulation research [22,41,54] that genotype-by-environment interactions are also important for maintaining stable intra-population mtDNA variation in nature [22,41] and compels for further research to be performed on this phenomenon.

Taken together, the fitness assays performed on *D. obscura* show the complexity of maintenance of sympatric mtDNA variation. Different balancing selection mechanisms may operate simultaneously in upholding joint genomic polymorphism in the same model. Our results give more weight to environment-mediated selection compared to SSS. However, the question stands as to what extent these results could be extrapolated to variation in natural habitats. Our experiment was performed on arbitrarily chosen temperatures, compared to the continual and unpredictable variation in nature. While sex is a discrete variable, the temperature is continuous, and different results could have been observed if fitness was compared at other experimental temperatures or other environmental conditions.

## Figures and Tables

**Table 1:** *D. obscura Cyt b* haplotypes used in the experiment (left) and their corresponding IFL names (right) per experimental block. ST, Tara Mountain; SS, Balkan Mountains; SG, Goč Mountain.

I EB Population ST Site1		II EB Population STSite2		III EB Population SS		IV EB Population SG	
O2	A	O9	D	O2	L	O2	O
O1	B	O2	E	O4	M	O3	P
O8	C	O10	F	O3	N	O18	Q

**Table 2:** The effect of mitochondrial haplotype (MT), nuclear genetic background (NU), sex, temperature (T) and their interactions on desiccation resistance for the experimental block (EB) I of *D. obscura*. LogLik, log-likelihood; Chisq, chi-squared value; Df, degrees of freedom; strata, variable is stratified; *p* values that are significant at *p* < 0.05 are given in bold. Reduced Cox proportional hazards models for pairwise comparisons with some interactions missing for this EB was presented at the 1st International Electronic Conference on Entomology, giving qualitatively indistinguishable results [57].

EB I	AB				AC				BC				ABC			
	LogLik	Chisq	Df	*p*	LogLik	Chisq	Df	*p*	LogLik	Chisq	Df	*p*	LogLik	Chisq	Df	*p*
MT	−2531.4	9.240	1	**0.0024**	−2966.2	13.56	1	**0.00023**	−2994.8	2.284	1	0.13072	−6307.2	5.74	2	0.05684
NU		strata			−2955.7	21.16	1	**4.2 × 10^−6^**		strata				strata		
sex	−2521.6	19.570	1	**9.7 × 10^−6^**	−2949.9	11.54	1	**0.00068**	−2989.2	11.348	1	**0.00076**		strata		
temp(T)		strata				strata			−2952.2	73.989	1	**2.2 × 10^−16^**	−6294.2	26.03	1	**3.4 × 10^−7^**
MT:NU	−2506.3	30.534	1	**3.3 × 10^−8^**	−2941.9	16.07	1	**6.1 × 10^−5^**	−2949.7	4.846	1	**0.02771**	−6264.9	58.51	4	**6.0 × 10^−12^**
MT:sex	−2503.6	1.352	1	0.2450	−2936.6	10.55	1	**0.00116**	−2943.6	12.341	1	**0.00044**	−6264	1.79	2	0.40841
NU:sex	−2503.5	0.104	1	0.7473	−2936.6	0.00	1	0.95096	−2942.6	1.997	1	0.15766		strata		
MT:T	−2504.2	4.209	1	**0.0402**	−2934	5.24	1	**0.02209**	−2938.1	8.856	1	**0.00292**	−6255.5	17.14	2	**0.00019**
NU:T		strata			−2934	0.01	1	0.93708	−2930.1	16.104	1	**6.0 × 10^−5^**	−6254.9	1.09	2	0.57892
T:sex	−2498.3	10.356	1	**0.0013**	−2927.2	13.43	1	**0.00025**	−2916.3	27.490	1	**1.6 × 10^−7^**	−6233	43.86	1	**3.5 × 10^−11^**
MT:NU:sex	−2477.9	0.056	1	0.8122	−2926.4	1.61	1	0.20438	−2916.3	0.004	1	0.94981	−6202.2	61.54	4	**1.4 × 10^−12^**
MT:NU:T	−2477.9	40.789	1	**1.7 × 10^−10^**	−2925.7	1.43	1	0.23108	−2913.7	5.255	1	**0.02188**	−6200.9	2.60	4	0.62690
MT:T:sex	−2476.7	2.378	1	0.1230	−2925.4	0.59	1	0.44138	−2912.2	3.115	1	0.07760	−6200.6	0.56	2	0.75397
NU:T:sex	−2476.6	0.151	1	0.6972	−2925.1	0.62	1	0.43070	−2911.7	0.856	1	0.35477	−6200.1	0.99	2	0.61096
MT:NU:T:sex	−2476.6	0.163	1	0.6866	−2925	0.16	1	0.69248	−2907.9	7.542	1	**0.00603**	−6194.1	12.01	4	**0.01725**

**Table 3:** The effect of mitochondrial haplotype (MT), nuclear genetic background (NU), sex, temperature (T) and their interactions on desiccation resistance for the experimental block (EB) II of *D. obscura*. LogLik, log-likelihood; Chisq, chi-squared value; Df, degrees of freedom; strata, variable is stratified; *p* values that are significant at *p* < 0.05 are given in bold.

EB II	DE				DF				EF				DEF			
	LogLik	Chisq	Df	*p*	LogLik	Chisq	Df	*p*	LogLik	Chisq	Df	*p*	LogLik	Chisq	Df	*p*
MT	−2993.6	4.84	1	**0.02777**	−2601.3	0.61	1	0.43587	−2964.5	28.42	1	**9.7 × 10^−8^**	−7897.5	53.47	2	**2.5 × 10^−12^**
NU	−2993.6	0.00	1	0.94573		strata			−2945.6	37.76	1	**8.0 × 10^−10^**	−7869.6	55.87	2	**7.4 × 10^−13^**
sex	−2992	3.24	1	0.07200	−2591.6	19.53	1	**9.9 × 10^−6^**	−2944.5	2.16	1	0.14148		strata		
temp(T)		strata				strata							−7860.8	17.61	1	**2.7 × 10^−5^**
MT:NU	−2991.1	1.74	1	0.18733	−2591	1.27	1	0.26037	−2926.3	36.45	1	**1.6 × 10^−9^**	−7840.7	40.11	4	**4.1 × 10^−8^**
MT:sex	−2988.3	5.54	1	**0.01859**	−2587.5	7.00	1	**0.00816**	−2926.3	0.01	1	0.90468	−7832.5	16.34	2	**0.00028**
NU:sex	−2973.7	29.23	1	**6.4 × 10^−8^**	−2584.9	5.02	1	**0.02511**	−2919.9	12.83	1	**0.00034**	-7818.8	27.50	2	**1.1 × 10^−6^**
MT:T	−2973.4	0.61	1	0.43353	−2580.8	8.23	1	**0.00412**	−2916.2	7.44	1	**0.00638**	−7812.6	12.39	2	**0.00204**
NU:T	−2963.6	19.60	1	**9.6 × 10^−6^**		strata			−2910	12.40	1	**0.00043**	−7790	45.24	2	**1.5 × 10^−10^**
T:sex	−2961.2	4.74	1	**0.02946**	−2578.8	3.96	1	**0.04666**	−2904.3	11.41	1	**0.00073**	−7781.2	17.49	1	**0.00003**
MT:NU:sex	−2961	0.45	1	0.50107	−2578.8	0.02	1	0.87763	−2904.3	0.01	1	0.92003	−7762.1	38.16	4	**1.0 × 10^−7^**
MT:NU:T	−2961	0.06	1	0.81429	−2577.1	3.57	1	0.05874	−2882.4	43.78	1	**3.7 × 10^−11^**	−7760.9	2.44	4	0.65468
MT:T:sex	−2960.6	0.84	1	0.35998	−2576.6	0.98	1	0.32177	−2882.1	0.53	1	0.46709	−7755.2	11.46	2	**0.00325**
NU:T:sex	−2954.1	12.92	1	**0.00032**	−2575.9	1.35	1	0.24562	−2878.8	6.58	1	**0.01032**	−7749.9	10.63	2	**0.00491**
MT:NU:T:sex	−2952.2	3.76	1	0.05246	−2574.6	2.65	1	0.10334	−2872.9	11.73	1	**0.00061**	−7738.2	23.39	4	**0.00011**

**Table 4:** The effect of mitochondrial haplotype (MT), nuclear genetic background (NU), sex, temperature (T) and their interactions on desiccation resistance for the experimental block (EB) III of *D. obscura*. LogLik, log-likelihood; Chisq, chi-squared value; Df, degrees of freedom; strata, variable is stratified; *p* values that are significant at *p* < 0.05 are given in bold. Reduced Cox proportional hazards models for pairwise comparisons with some interactions missing for this EB was presented at the 1st International Electronic Conference on Entomology, giving qualitatively indistinguishable results [57].

EB III	LM				LN				MN				LMN			
	LogLik	Chisq	Df	*p*	LogLik	Chisq	Df	*p*	LogLik	Chisq	Df	*p*	LogLik	Chisq	Df	*p*
MT	−2873.1	2.08	1	0.14912	−3278.7	4.07	1	**0.04362**	−2798.6	**0.09**	1	0.76424	−6391.6	5.60	2	0.06090
NU	−2853.4	39.27	1	**3.7 × 10^−10^**	−3219	119.33	1	**2.2 × 10^−16^**	−2780.6	**36.17**	1	**1.8 × 10^−9^**		strata		
sex	−2851.2	4.48	1	**0.03431**	−3218.3	1.44	1	0.23044	−2779.1	**2.94**	1	0.08623	−6295.7	191.83	1	**2.2 × 10^−16^**
temp(T)	−2769	164.44	1	**2.2 × 10^−16^**	−3149.4	137.81	1	**2.2 × 10^−16^**	−2761.2	**35.71**	1	**2.3 × 10^−9^**	−6295.6	0.15	1	0.70212
MT:NU	−2764.4	9.08	1	**0.00259**	−3144.6	9.59	1	**0.00195**	−2756.8	**8.74**	1	**0.00312**	−6271.9	47.28	4	**1.3 × 10^−9^**
MT:sex	−2764.4	0.03	1	0.86929	−3142.3	4.55	1	**0.03293**	−2756.8	**0.06**	1	0.80429	−6263.5	16.84	2	**0.00022**
NU:sex	−2761.3	6.26	1	**0.01236**	−3137.7	9.26	1	**0.00234**	−2755	**3.65**	1	0.05622	−6246.6	33.81	2	**4.6 × 10^−8^**
MT:T	−2754.5	13.65	1	**0.00022**	−3134.7	6.01	1	**0.01423**	−2752	**5.98**	1	**0.01446**	−6245.2	2.87	2	0.23812
NU:T	−2752.1	4.69	1	**0.03030**	−3131.8	5.67	1	**0.01723**	−2751.9	0.23	1	0.63140	−6237.3	15.86	2	**0.00036**
T:sex	−2752.1	0.03	1	0.85993	−3129.3	5.11	1	**0.02385**	−2750.3	**3.26**	1	0.07112	−6235.9	2.68	1	0.10149
MT:NU:sex	−2751.9	0.45	1	0.50464	−3127.3	3.92	1	**0.04782**	−2750.1	0.42	1	0.51560	−6209.7	52.40	4	**1.1 × 10^−10^**
MT:NU:T	−2749.1	5.48	1	**0.01923**	−3123.6	7.48	1	**0.00625**	−2737.8	24.51	1	**7.4 × 10^−7^**	−6205.7	7.96	4	0.09311
MT:T:sex	−2748.8	0.69	1	0.40653	−3117	13.18	1	**0.00028**	−2731.5	12.62	1	**0.00038**	−6196.4	18.62	2	**9.1 × 10^−5^**
NU:T:sex	−2747.1	3.43	1	0.06399	−3116.9	0.17	1	0.67903	−2730.3	2.45	1	0.11786	−6195.3	2.18	2	0.33614
MT:NU:T:sex	−2746.7	0.72	1	0.39545	−3114.6	4.54	1	**0.03320**	−2729.8	0.98	1	0.32162	−6192.6	5.49	4	0.24058

**Table 5:** The effect of mitochondrial haplotype (MT), nuclear genetic background (NU), sex, temperature (T) and their interactions on desiccation resistance for the experimental block (EB) II of *D. obscura*. LogLik, log-likelihood; Chisq, chi-squared value; Df, degrees of freedom; strata, variable is stratified; *p* values that are significant at *p* < 0.05 are given in bold.

EB IV	OP				OQ				PQ				OPQ			
	LogLik	Chisq	Df	*p*	LogLik	Chisq	Df	*p*	LogLik	Chisq	Df	*p*	LogLik	Chisq	Df	*p*
MT	−2915.2	0.86	1	0.35422	−2983.5	2.02	1	0.15542	−2976.2	5.10	1	0.02397	−6261.5	10.45	2	**0.00537**
NU	−2915.2	0.02	1	0.88632		strata				strata				strata		
sex		strata			−2972.6	21.85	1	**2.9 × 10^−6^**	−2976.1	0.07	1	0.79310	−6050.6	421.85	1	**2.2 × 10^−16^**
temp(T)	−2810.4	209.51	1	**2.2 × 10^−16^**	−2824.3	296.56	1	**2.2 × 10^−16^**	−2888.9	174.40	1	**2.2 × 10^−16^**		strata		
MT:NU	−2809.9	0.99	1	0.32078	−2824.3	0.00	1	0.96923	−2882.8	12.31	1	**0.00045**	−6042.6	16.07	4	**0.00293**
MT:sex	−2801.8	16.30	1	**5.4 × 10^−5^**	−2822.7	3.21	1	0.07312	−2879.1	7.36	1	**0.00667**	−6034.5	16.05	2	**0.00033**
NU:sex	−2801.8	0.01	1	0.93663	−2818.3	8.78	1	**0.00304**	−2879.1	0.07	1	0.79796	−6032.6	3.96	2	0.13834
MT:T	−2800.4	2.72	1	0.09938	−2816.2	4.26	1	**0.03900**	−2879	0.06	1	0.80007	−6017.3	30.56	2	**2.3 × 10^−7^**
NU:T	−2800.4	0.01	1	0.91035	−2815	2.31	1	0.12894	−2878.2	1.75	1	0.18603		strata		
T:sex	−2797.4	5.96	1	**0.01462**	−2814.6	0.80	1	0.36981	−2878	0.35	1	0.55487	−6015.4	3.78	1	0.05190
MT:NU:sex	−2796.4	2.03	1	0.15397	−2814.6	0.07	1	0.79290	−2874.8	6.38	1	**0.01152**	−6005.6	19.63	4	**0.00059**
MT:NU:T	−2795.5	1.87	1	0.17163	−2810.6	8.00	1	**0.00469**	−2864.6	20.40	1	**6.3 × 10^−6^**	−5998.3	14.43	4	**0.00603**
MT:T:sex	−2788.6	13.76	1	**0.00021**	−2805.3	10.55	1	**0.00116**	−2864.6	0.06	1	0.81162	−5990.1	16.49	2	**0.00026**
NU:T:sex	−2785.2	6.81	1	**0.00907**	−2798.7	13.27	1	**0.00027**	−2862.5	4.16	1	**0.04134**	−5969.5	41.23	2	**1.1 × 10^−9^**
MT:NU:T:sex	−2781.5	7.34	1	**0.00674**	−2794.8	7.64	1	**0.00570**	−2861.4	2.21	1	0.13668	−5962.8	13.45	4	**0.00929**

**Table 6:** The effect of mitochondrial haplotype (MT), nuclear genetic background (NU), sex, temperature (T) and their interactions on developmental time for the three pairwise comparisons from experimental block (EB) I and the whole EB I of *D. obscura*. SSq, sum of squares; ddf, denominator degrees of freedom; *p* values that are significant at *p* < 0.05 are given in bold.

EB I	AB				AC				BC				ABC			
	SSq	ddf	F	*p*	SSq	ddf	F	*p*	SSq	ddf	F	*p*	SSq	ddf	F	*p*
MT	0.450	70.0	0.62	0.4320	5.2	72.3	6.46	**0.0132**	0.1	69.6	0.10	0.7585	0.5	158.7	0.31	0.7374
NU	0.350	70.0	0.49	0.4870	7.8	72.3	9.61	**0.0028**	18.3	69.6	27.55	**1.6 × 10^−6^**	33.6	158.6	21.55	**5.3 × 10^−9^**
sex	5.400	931.4	7.43	**0.0065**	3.8	1042.2	4.67	**0.0309**	1.4	1028.8	2.18	0.1405	6.3	2214.3	8.12	**0.0044**
temp(T)	1726	70.0	2374	**2.2 × 10^−16^**	1675	72.3	2073	**2.2 × 10^−16^**	1353	69.6	2041	**2.2 × 10^−16^**	3835	158.7	4913	**2.2 × 10^−16^**
MT:NU	3.120	70.0	4.30	**0.0419**	5.3	72.3	6.50	**0.0129**	1.5	69.6	2.20	0.1428	14.4	158.5	4.63	**0.0015**
MT:sex	0.210	931.4	0.29	0.5928	1.3	1042.2	1.56	0.2123	1.1	1028.8	1.73	0.1892	1	2214.2	0.65	0.5217
NU:sex	1.670	931.4	2.30	0.1298	2.1	1042.2	2.56	0.1100	0.5	1028.8	0.68	0.4113	2.5	2213.9	1.60	0.2019
MT:T	1.740	70.0	2.39	0.1263	37.4	72.3	46.29	**2.5 × 10^−9^**	0.1	69.6	0.13	0.7176	5.1	158.7	3.25	**0.0416**
NU:T	0.950	70.0	1.31	0.2571	21.0	72.3	26.04	**2.6 × 10^−6^**	7.4	69.6	11.12	**0.0014**	34.9	158.6	22.35	**2.8 × 10^−9^**
T:sex	0.490	931.4	0.67	0.4131	3.7	1042.2	4.53	**0.0336**	5.6	1028.8	8.42	**0.0038**	4.6	2214.3	5.95	**0.0148**
MT:NU:sex	1.550	931.4	2.14	0.1441	0.0	1042.2	0.01	0.9115	0.0	1028.8	0.01	0.9201	1.9	2213.6	0.60	0.6591
MT:NU:T	14.96	70.0	20.58	**2.3 × 10^−5^**	0.2	72.3	0.24	0.6277	3.9	69.6	5.84	**0.0183**	55.4	158.5	17.75	**4.6 × 10^−12^**
MT:T:sex	2.310	931.4	3.18	0.0749	0.0	1042.2	0.00	0.9446	1.2	1028.8	1.75	0.1857	0.3	2214.2	0.20	0.8185
NU:T:sex	1.810	931.4	2.49	0.1146	0.0	1042.2	0.01	0.9263	1.9	1028.8	2.91	0.0885	3	2213.9	1.93	0.1452
MT:NU:T:sex	1.990	931.4	2.74	0.0984	0.2	1042.2	0.25	0.6154	0.0	1028.8	0.05	0.8181	7.6	2213.6	2.44	**0.0446**

**Table 7:** The effect of mitochondrial haplotype (MT), nuclear genetic background (NU), sex, temperature (T) and their interactions on developmental time for the three pairwise comparisons from experimental block (EB) II and the whole EB II of *D. obscura*. SSq—sum of squares; ddf, denominator degrees of freedom; *p* values that are significant at *p* < 0.05 are given in bold.

EB II	DE				DF				EF				DEF			
	SSq	ddf	F	*p*	SSq	ddf	F	*p*	SSq	ddf	F	*p*	SSq	ddf	F	*p*
MT	5.54	70.66	5.63	**0.020**	9.28	70.7	8.72	**0.0043**	32.1	71.14	33.26	**1.9 × 10^−7^**	45.3	160.0	22.66	**2.2 × 10^−9^**
NU	3.93	70.66	4.00	**0.049**	20.78	70.7	19.52	**3.5 × 10^−5^**	3.9	71.14	4.07	**0.0474**	12.7	160.0	6.33	**0.0023**
sex	6.36	954.08	6.46	**0.011**	0.25	996.9	0.24	0.6271	0.9	996	0.91	0.3396	3.7	2233.5	3.72	0.0538
temp(T)	2474	70.66	2514	**2 × 10^−16^**	3028	72.0	2845	**2.2 × 10^−16^**	3265	71.14	3378	**2.2 × 10^−16^**	6008	160.1	6008	**2.2 × 10^−16^**
MT:NU	0	70.66	0.00	0.982	0.08	70.7	0.08	0.7843	8.3	71.14	8.64	**0.0044**	8.2	160.0	2.06	0.0889
MT:sex	1.72	954.08	1.75	0.186	3.69	996.9	3.46	0.0631	6.5	996	6.71	**0.0097**	5.8	2233.1	2.90	0.0554
NU:sex	3.57	954.08	3.63	0.057	2.36	996.9	2.21	0.1371	1.6	996	1.67	0.1960	14.6	2233.0	7.28	**0.0007**
MT:T	0.96	70.66	0.98	0.326	6.81	72.0	6.40	**0.0136**	14.6	71.14	15.15	**0.0002**	19.6	160.0	9.81	**9.5 × 10^−5^**
NU:T	1.77	70.66	1.80	0.184	26.3	72.0	24.71	**4.4 × 10^−6^**	15.4	71.14	15.95	**0.0002**	38.4	160.0	19.20	**3.4 × 10^−8^**
T:sex	1.6	954.08	1.63	0.202	0.51	999.3	0.48	0.4875	0	996	0.02	0.8837	2.2	2233.5	2.20	0.1382
MT:NU:sex	1.38	954.08	1.41	0.236	0.05	996.9	0.04	0.8339	0.2	996	0.21	0.6484	5.2	2231.9	1.31	0.2643
MT:NU:T	0.05	70.66	0.05	0.826	0.09	72.0	0.08	0.7771	0.3	71.14	0.32	0.5719	0.5	160.0	0.14	0.9685
MT:T:sex	1.52	954.08	1.54	0.215	3.48	999.3	3.27	0.0710	4.9	996	5.03	**0.0251**	3.4	2233.1	1.69	0.1850
NU:T:sex	3.33	954.08	3.38	0.066	3.42	999.3	3.21	0.0735	7.4	996	7.66	**0.0057**	7.2	2233.0	3.62	**0.0269**
MT:NU:T:sex	0.01	954.08	0.01	0.943	0	999.3	0.00	0.9865	6.5	996	6.77	**0.0094**	12.2	2231.9	3.05	**0.0162**

**Table 8:** The effect of mitochondrial haplotype (MT), nuclear genetic background (NU), sex, temperature (T) and their interactions on developmental time for the three pairwise comparisons from experimental block (EB) III and the whole EB III of *D. obscura*. SSq, sum of squares; ddf, denominator degrees of freedom; *p* values that are significant at *p* < 0.05 are given in bold.

EB III	LM				LN				MN				LMN			
	SSq	ddf	F	*p*	SSq	ddf	F	*p*	SSq	ddf	F	*p*	SSq	ddf	F	*p*
MT	0.08	352.01	0.08	0.774	2.56	79.85	1.64	0.2040	10.17	263.43	10.84	**0.0011**	1.13	508.12	0.48	0.6201
NU	0.08	352.01	0.08	0.772	0.77	79.85	0.50	0.4832	19.82	263.43	21.13	**6.7** **× 10^−6^**	20.16	350.54	8.55	**0.0002**
sex	0.68	684.68	0.75	0.388	4.85	623.7	3.11	0.0783	0.35	782.68	0.37	0.5435	5.25	1563.83	4.45	**0.0350**
temp(T)	840	352.01	917	**2** **× 10^−16^**	2147	79.85	1376	**2** **× 10^−16^**	1447	263.43	1543	**2.2** **× 10^−16^**	2672	550.98	2267	**2.2** **× 10^−16^**
MT:NU	2.89	352.01	3.15	0.077	0.84	79.85	0.54	0.4657	9.71	263.43	10.35	**0.0015**	23.37	334.23	4.96	**0.0007**
MT:sex	0.41	684.68	0.44	0.506	0.03	623.7	0.02	0.8810	1.43	782.68	1.52	0.2174	0.09	1566.61	0.04	0.9619
NU:sex	2.61	684.68	2.84	0.092	0	623.7	0.00	0.9913	2.93	782.68	3.12	0.0776	1.56	1582.77	0.66	0.5158
MT:T	0.11	352.01	0.12	0.731	5.7	79.85	3.65	0.0595	2.61	263.43	2.78	0.0968	1.05	508.12	0.45	0.6408
NU:T	0.19	352.01	0.20	0.653	6.99	79.85	4.48	**0.0375**	11.09	263.43	11.82	**0.0007**	5.67	350.54	2.41	0.0917
T:sex	0.31	684.68	0.34	0.558	0.77	623.7	0.49	0.4830	2.09	782.68	2.23	0.1356	0.14	1563.83	0.11	0.7348
MT:NU:sex	4.55	684.68	4.97	**0.026**	0.06	623.7	0.04	0.8425	5.77	782.68	6.15	**0.0134**	8.51	1580.6	1.81	0.1252
MT:NU:T	8.14	352.01	8.89	**0.003**	0.6	79.85	0.39	0.5365	0	263.43	0.00	0.9458	10.77	334.23	2.29	0.0600
MT:T:sex	2.8	684.68	3.06	0.081	1.96	623.7	1.26	0.2626	0.07	782.68	0.07	0.7913	3.37	1566.61	1.43	0.2395
NU:T:sex	0.08	684.68	0.09	0.770	2.56	623.7	1.64	0.2009	0.31	782.68	0.33	0.5684	1.94	1582.77	0.82	0.4396
MT:NU:T:sex	1.14	684.68	1.25	0.265	0.39	623.7	0.25	0.6165	0.96	782.68	1.02	0.3117	2.41	1580.6	0.51	0.7280

**Table 9:** The effect of mitochondrial haplotype (MT), nuclear genetic background (NU), sex, temperature (T) and their interactions on developmental time for the three pairwise comparisons from experimental block (EB) IV and the whole EB IV of *D. obscura*. SSq, sum of squares; ddf, denominator degrees of freedom; *p* values that are significant at *p* < 0.05 are given in bold.

EB IV	OP				OQ				PQ				OPQ			
	SSq	ddf	F	*p*	SSq	ddf	F	*p*	SSq	ddf	F	*p*	SSq	ddf	F	*p*
MT	4.32	74.1	5.23	**0.02510**	3.07	82.0	2.99	0.0877	28.92	66.4	45.15	**5.0** **× 10^−9^**	22.88	172.1	14.38	**1.7** **× 10^−6^**
NU	25.84	74.1	31.28	**3.6** **× 10^−7^**	2.46	82.0	2.39	0.1263	0.73	66.4	1.15	0.2881	19.83	171.9	12.47	**8.8** **× 10^−6^**
sex	9.05	1146	10.95	**0.00097**	20.83	884.2	20.23	**7.8** **× 10^−6^**	9.46	1014	14.76	**0.0001**	29.81	2318	37.47	**1.1** **× 10^−9^**
temp(T)	1690	74.1	2045	**2.2** **× 10^−16^**	1235	82.0	1199	**2.2** **× 10^−16^**	1251	66.4	1953	**2.2** **× 10^−16^**	3075	172.6	3865	**2.2** **× 10^−16^**
MT:NU	3.18	74.1	3.85	0.05362	23.28	82.0	22.61	**8.4** **× 10^−6^**	9.90	66.4	15.46	**0.0002**	28.23	171.0	8.87	**1.6** **× 10^−6^**
MT:sex	0.01	1146	0.01	0.91750	0.08	884.2	0.07	0.7870	2.70	1014	4.21	**0.0404**	2.04	2315	1.28	0.2779
NU:sex	6.47	1146	7.82	**0.00524**	0.10	884.2	0.10	0.7572	0.89	1014	1.39	0.2392	6.15	2314	3.87	**0.0211**
MT:T	2.19	74.1	2.64	0.10813	0.00	82.0	0.00	0.9813	0.17	66.4	0.27	0.6049	2.55	172.1	1.60	0.2045
NU:T	5.55	74.1	6.72	**0.01149**	0.08	82.0	0.08	0.7833	8.87	66.4	13.84	**0.0004**	18.35	171.9	11.53	**2.0** **× 10^−5^**
T:sex	0.15	1146	0.18	0.66836	0.20	884.2	0.20	0.6581	0.12	1014	0.18	0.6711	0.38	2318	0.48	0.4905
MT:NU:sex	0.03	1146	0.04	0.83715	0.85	884.2	0.82	0.3643	0.10	1014	0.16	0.6907	2.65	2311	0.83	0.5049
MT:NU:T	2.1	74.1	2.55	0.11480	0.00	82.0	0.00	0.9927	0.23	66.4	0.36	0.5500	2.12	171.0	0.67	0.6167
MT:T:sex	1.21	1146	1.47	0.22586	0.41	884.2	0.40	0.5279	0.71	1014	1.11	0.2914	0.52	2315	0.33	0.7202
NU:T:sex	0.15	1146	0.18	0.66802	2.99	884.2	2.90	0.0888	0.03	1014	0.05	0.8156	1.03	2314	0.65	0.5230
MT:NU:T:sex	6.44	1146	7.79	**0.00534**	0.02	884.2	0.02	0.8987	0.11	1014	0.18	0.6740	7.18	2311	2.26	0.0607

**Table 10:** The effect of mitochondrial haplotype (MT), nuclear genetic background (NU), temperature (T) and their interactions on Egg-to-adult viability for three pairwise comparisons and the experimental block (EB) analysed as a whole for each of the four experimental blocks. DF, degrees of freedom; Dev, deviance; *p* values that are significant at *p* < 0.05 are given in bold.

**EB I**	**AB**			**AC**			**BC**			**ABC**		
	**Df**	**Dev**	***p***	**Df**	**Dev**	***p***	**Df**	**Dev**	***p***	**Df**	**Dev**	***p***
MT	1	1.11	0.2932	1	7.24	**0.0071**	1	1.874	0.1711	2	7.041	**0.0296**
NU	1	40.66	**1.8** **× 10^−10^**	1	132.6	**2.2** **× 10^−16^**	1	171.0	**2.2** **× 10^−16^**	2	288.7	**2.2** **× 10^−16^**
temp(T)	1	0.18	0.6732	1	20.15	**7.2** **× 10^−6^**	1	57.03	**4.3** **× 10^−14^**	1	48.41	**3.5** **× 10^−12^**
MT:NU	1	17.38	**3.1** **× 10^−5^**	1	78.50	**2.2** **× 10^−16^**	1	2.71	0.0997	4	101.2	**2.2** **× 10^−16^**
MT:T	1	18.39	**1.8** **× 10^−5^**	1	1.45	0.2285	1	15.77	**7.1** **× 10^−5^**	2	16.51	**2.6** **× 10^−4^**
NU:T	1	3.23	0.0723	1	70.44	**2.2** **× 10^−16^**	1	15.24	**9.5** **× 10^−5^**	2	62.91	**2.2** **× 10^−14^**
MT:NU:T	1	11.82	**0.0006**	1	1.839	0.1751	1	2.761	0.0966	4	40.30	**3.7** **× 10^−8^**
**EB II**	**DE**			**DF**			**EF**			**DEF**		
	**Df**	**Dev**	***p***	**Df**	**Dev**	***p***	**Df**	**Dev**	***p***	**Df**	**Dev**	***p***
MT	1	15.51	**8.2** **× 10^−5^**	1	10.55	**0.0012**	1	1.458	0.2273	2	11.51	**0.0032**
NU	1	66.97	**2.8** **× 10^−16^**	1	34.21	**4.9** **× 10^−9^**	1	35.74	**2.3** **× 10^−9^**	2	130.5	**2.2** **× 10^−16^**
temp(T)	1	29.32	**6.1** **× 10^−8^**	1	87.20	**2.2** **× 10^−16^**	1	40.59	**1.9** **× 10^−10^**	1	200.5	**2.2** **× 10^−16^**
MT:NU	1	2.596	0.1071	1	23.32	**1.4** **× 10^−6^**	1	13.11	**0.0003**	4	76.41	**1.0** **× 10^−15^**
MT:T	1	31.34	**2.2** **× 10^−8^**	1	9.333	**0.0023**	1	19.69	**9.1** **× 10^−6^**	2	23.56	**7.7** **× 10^−6^**
NU:T	1	0.811	0.3678	1	19.71	**9.0** **× 10^−6^**	1	62.83	**2.3** **× 10^−15^**	2	31.95	**1.2** **× 10^−7^**
MT:NU:T	1	149.9	**2.2** **× 10^−16^**	1	15.07	**0.0001**	1	36.52	**1.5** **× 10^−9^**	4	173.4	**2.2** **× 10^−16^**
**EB III**	**LM**			**LN**			**MN**			**LMN**		
	**Df**	**Dev**	***p***	**Df**	**Dev**	***p***	**Df**	**Dev**	***p***	**Df**	**Dev**	***p***
MT	1	1.085	0.2976	1	28.27	**1.1** **× 10^−7^**	1	6.41	**0.0114**	2	18.83	**8.1** **× 10^−5^**
NU	1	238.3	**2.2** **× 10^−16^**	1	56.36	**6.0** **× 10^−14^**	1	94.99	**2.2** **× 10^−16^**	2	522.3	**2.2** **× 10^−16^**
temp(T)	1	313.8	**2.2** **× 10^−16^**	1	18.26	**1.9** **× 10^−5^**	1	225.9	**2.2** **× 10^−16^**	1	266.4	**2.2** **× 10^−16^**
MT:NU	1	8.779	**0.0030**	1	17.70	**2.6** **× 10^−5^**	1	16.53	**4.8** **× 10^−5^**	4	55.97	**2.0** **× 10^−11^**
MT:T	1	32.16	**1.4** **× 10^−8^**	1	27.78	**1.4** **× 10^−7^**	1	1.581	0.2087	2	37.28	**8.0** **× 10^−9^**
NU:T	1	0.115	0.7341	1	2.159	0.1418	1	125.2	**2.2** **× 10^−16^**	2	106.3	**2.2** **× 10^−16^**
MT:NU:T	1	28.06	**1.2** **× 10^−7^**	1	50.24	**1.4** **× 10^−12^**	1	1.9	0.1681	4	126.0	**2.2** **× 10^−16^**
**EB IV**	**OP**			**OQ**			**PQ**			**OPQ**		
	**Df**	**Dev**	***p***	**Df**	**Dev**	***p***	**Df**	**Dev**	***p***	**Df**	**Dev**	***p***
MT	1	20.38	**6.3** **× 10^−6^**	1	78.53	**2.2** **× 10^−16^**	1	87.32	**2.0** **× 10^−16^**	2	200.8	**2.2** **× 10^−16^**
NU	1	9.33	**0.0023**	1	103.0	**2.2** **× 10^−16^**	1	327.1	**2.0** **× 10^−16^**	2	447.3	**2.2** **× 10^−16^**
temp(T)	1	45.95	**1.2** **× 10^−11^**	1	2.752	0.0971	1	0	0.9581	1	17.14	**3.5** **× 10^−5^**
MT:NU	1	7.112	**0.0077**	1	0.01	0.9197	1	128.7	**2.0** **× 10^−16^**	4	148.3	**2.2** **× 10^−16^**
MT:T	1	0.061	0.8051	1	57.04	**4.3** **× 10^−14^**	1	1.42	0.2339	2	61.24	**5.0** **× 10^−14^**
NU:T	1	0.299	0.5844	1	6.415	**0.0113**	1	5.57	**0.0183**	2	2.32	0.3130
MT:NU:T	1	2.017	0.1555	1	0.134	0.7144	1	0.48	0.4900	4	23.16	**0.0001**

**Table 11:** The effect of mitochondrial haplotype (MT), nuclear genetic background (NU), temperature (T) and their interactions on the percentage of males for three pairwise comparisons and the experimental block (EB) analysed as a whole for each of the four experimental blocks. DF, degrees of freedom; Dev, deviance; *p* values that are significant at *p* < 0.05 are given in bold.

**EB I**	**AB**			**AC**			**BC**			**ABC**		
	**Df**	**Dev**	***p***	**Df**	**Dev**	***p***	**Df**	**Dev**	***p***	**Df**	**Dev**	***p***
MT	1	0.02	0.8808	1	0.04739	0.8277	1	1.33724	0.24750	2	0.36	0.834
NU	1	0.00	0.9872	1	2.74073	0.0978	1	0.00149	0.96920	2	1.12	0.571
temp(T)	1	0.30	0.5828	1	0.09581	0.7569	1	0.00376	0.95110	1	0.24	0.626
MT:NU	1	0.01	0.9396	1	0.48097	0.4880	1	0.30348	0.58170	4	2.47	0.651
MT:T	1	0.09	0.7636	1	1.0408	0.3076	1	0.0083	0.92740	2	0.16	0.922
NU:T	1	1.78	0.1825	1	0.48283	0.4871	1	0.67503	0.41130	2	0.35	0.841
MT:NU:T	1	1.92	0.1659	1	0.06717	0.7955	1	1.32005	0.25060	4	5.53	0.237
**EB II**	**DE**			**DF**			**EF**			**DEF**		
	**Df**	**Dev**	***p***	**Df**	**Dev**	***p***	**Df**	**Dev**	***p***	**Df**	**Dev**	***p***
MT	1	4.51	**0.03379**	1	1.6082	0.20470	1	0.1452	0.70314	2	12.25	**0.002**
NU	1	0.62	0.43183	1	0.1475	0.70100	1	2.5504	0.11027	2	28.76	**6** **× 10^−7^**
temp(T)	1	7.31	**0.00686**	1	1.6117	0.20430	1	15.36	**8.9** **× 10^−5^**	1	0.31	0.578
MT:NU	1	1.09	0.29647	1	0.0122	0.91210	1	4.9746	**0.02572**	4	19.09	**0.001**
MT:T	1	4.97	**0.02586**	1	15.2255	**9.5** **× 10^−5^**	1	0.5121	0.47425	2	0.74	0.691
NU:T	1	8.00	**0.00468**	1	0.2847	0.59360	1	10.6933	**0.00108**	2	5.59	0.061
MT:NU:T	1	1.10	0.29332	1	0.5458	0.46000	1	0.3765	0.53949	4	6.17	0.187
**EB III**	**LM**			**LN**			**MN**			**LMN**		
	**Df**	**Dev**	***p***	**Df**	**Dev**	***p***	**Df**	**Dev**	***p***	**Df**	**Dev**	***p***
MT	1	0.00	0.97074	1	0.3288	0.56638	1	12.92	**0.00032**	2	11.7	**0.003**
NU	1	472.89	**2.2** **× 10^−16^**	1	0.0007	0.97957	1	633.78	**2.2** **× 10^−16^**	2	1125.3	**2** **× 10^−16^**
temp(T)	1	8.40	**0.00375**	1	2.7945	0.09459	1	4.59	**0.03210**	1	5.5	**0.019**
MT:NU	1	15.68	**7.5** **× 10^−5^**	1	3.479	0.06215	1	23.35	**1.4** **× 10^−6^**	4	33.1	**1** **× 10^−6^**
MT:T	1	0.35	0.55523	1	0.8008	0.37085	1	3.57	0.05889	2	1.0	0.610
NU:T	1	8.54	**0.00348**	1	1.3167	0.25119	1	13.88	**0.00019**	2	9.3	**0.010**
MT:NU:T	1	14.50	**0.00014**	1	0.0018	0.96654	1	5.64	**0.01751**	4	16.9	**0.002**
**EB IV**	**OP**			**OQ**			**PQ**			**OPQ**		
	**Df**	**Dev**	***p***	**Df**	**Dev**	***p***	**Df**	**Dev**	***p***	**Df**	**Dev**	***p***
MT	1	35.30	**2.8** **× 10^−9^**	1	3.409	0.06486	1	0.0482	0.82617	2	2.20	0.334
NU	1	90.58	**2.2** **× 10^−16^**	1	7.96	**0.00478**	1	0.4858	0.48582	2	188.33	**2** **× 10^−16^**
temp(T)	1	1.13	0.28824	1	2.405	0.12093	1	0.5486	0.45890	1	0.68	0.409
MT:NU	1	66.80	**3.0** **× 10^−16^**	1	102.83	**2.2** **× 10^−16^**	1	0.9611	0.32692	4	168.34	**2** **× 10^−16^**
MT:T	1	39.46	**3.3** **× 10^−10^**	1	44.23	**2.9** **× 10^−11^**	1	0.1021	0.74928	2	27.32	**1** **× 10^−6^**
NU:T	1	0.48	0.49005	1	140.156	**2.2** **× 10^−16^**	1	4.3152	**0.03777**	2	31.88	**1** **× 10^−7^**
MT:NU:T	1	11.23	**0.00081**	1	0.674	0.41178	1	3.7143	0.05395	4	169.68	**2** **× 10^−16^**

## Data Availability

Raw data is provided in spreadsheet, and can be downloaded at supplementary materials.

## Supplementary Materials

The following supporting information can be downloaded at: https://www.mdpi.com/article/10.3390/insects13020139/s1, Figure S1: Mean survival times in hours for the desiccation experiment for all combinations of genotypes and sex from the EB I on two experimental temperatures. Figure S2: Mean survival times in hours for the desiccation experiment for all combinations of genotypes and sex from the EB II on two experimental temperatures. Figure S3: Mean survival times in hours for the desiccation experiment for all combinations of genotypes and sex from the EB III on two experimental temperatures. Figure S4: Mean survival times in hours for the desiccation experiment for all combinations of genotypes and sex from the EB IV on two experimental temperatures. Figure S5: Mean developmental times in days for all combinations of genotypes and sex from the EB I on two experimental temperatures. Figure S6: Mean developmental times in days for all combinations of genotypes and sex from the EB II on two experimental temperatures. Figure S7: Mean developmental times in days for all combinations of genotypes and sex from the EB III on two experimental temperatures. Figure S8: Mean developmental times in days for all combinations of genotypes and sex from the EB IV on two experimental temperatures. Figure S9: Mean egg-to-adult viability scores for all combinations of genotypes from the EB I on two experimental temperatures. Figure S10: Mean egg-to-adult viability scores for all combinations of genotypes from the EB II on two experimental temperatures. Figure S11: Mean egg-to-adult viability scores for all combinations of genotypes from the EB III on two experimental temperatures. Figure S12: Mean egg-to-adult viability scores for all combinations of genotypes from the EB IV on two experimental temperatures. Figure S13: Mean proportion of males for all combinations of genotypes from EB I on two experimental temperatures. Figure S14: Mean proportion of males for all combinations of genotypes from EB II on two experimental temperatures. Figure S15: Mean proportion of males for all combinations of genotypes from EB III on two experimental temperatures. Figure S16: Mean proportion of males for all combinations of genotypes from EB IV on two experimental temperatures. Table S1: The effect of mitochondrial haplotype (MT), nuclear genetic background (NU), sex, temperature (T) and their interactions on egg-to-pupa viability (EtP) for three pairwise comparisons and the experimental block (EB) analysed as a whole for each of the four experimental blocks. Table S2: The effect of mitochondrial haplotype (MT), nuclear genetic background (NU), sex, temperature (T) and their interactions on pupa-to-adult viability (PtA) for three pairwise comparisons and the experimental block (EB) analysed as a whole for each of the four experimental blocks.
