# Supplementary material for: Temperature-Specific and Sex-Specific Fitness Effects of Sympatric Mitochondrial and Mito-Nuclear Variation in Drosophila obscura
Source: Insects. 2022 Jan 28;13(2):139. doi: 10.3390/insects13020139 (PMC8880146; doi:10.3390/insects13020139)
Supplement: Supplementary file 1 [file insects-13-00139-s001.zip › Supplementary Figures and Legends.pdf]

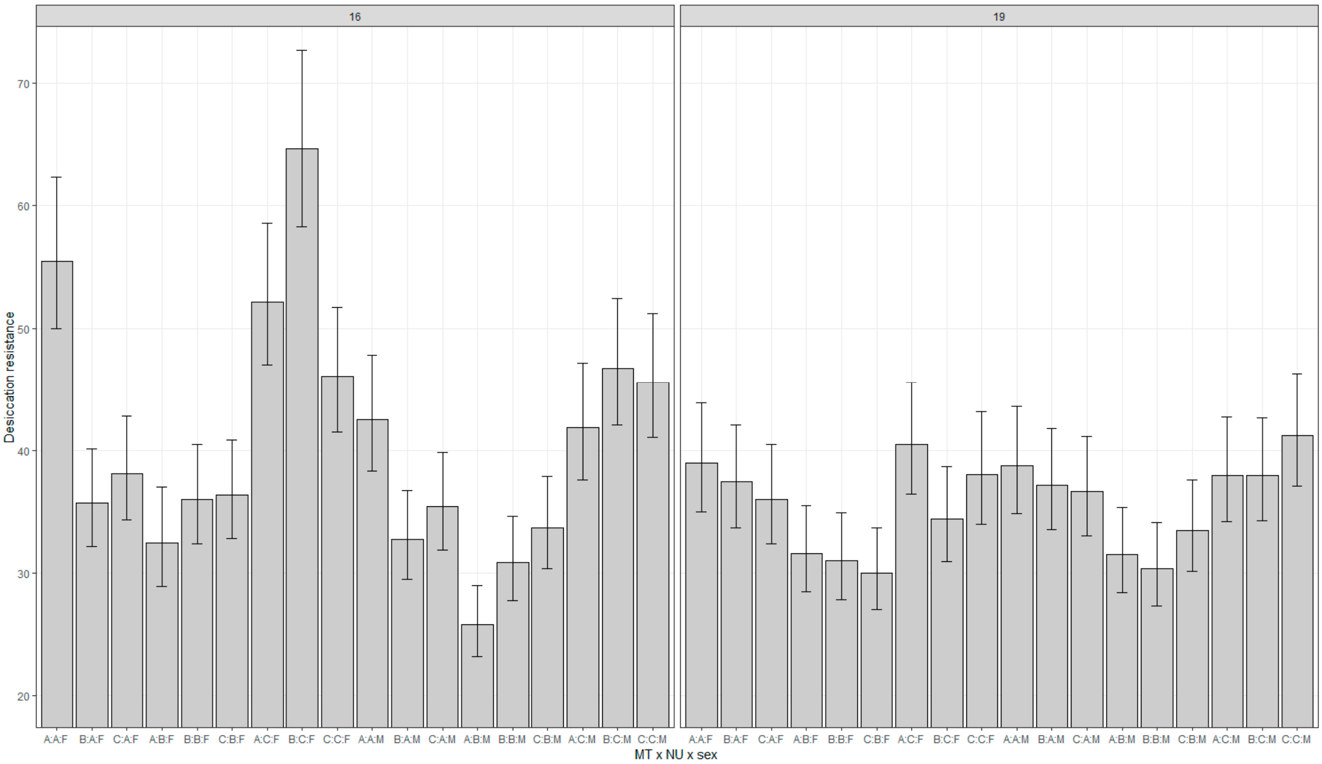

Supplementary Figure S1. Mean survival times in hours for the desiccation experiment for all combinations of genotypes and sex from the EB I on two experimental temperatures. A, B and C denote MNILs (the first letter marks mtDNA haplotype (MT), the second letter marks nuDNA background (NU)), F and M denote females and males, respectively (SEX). Error bars represent 95% confidence intervals.

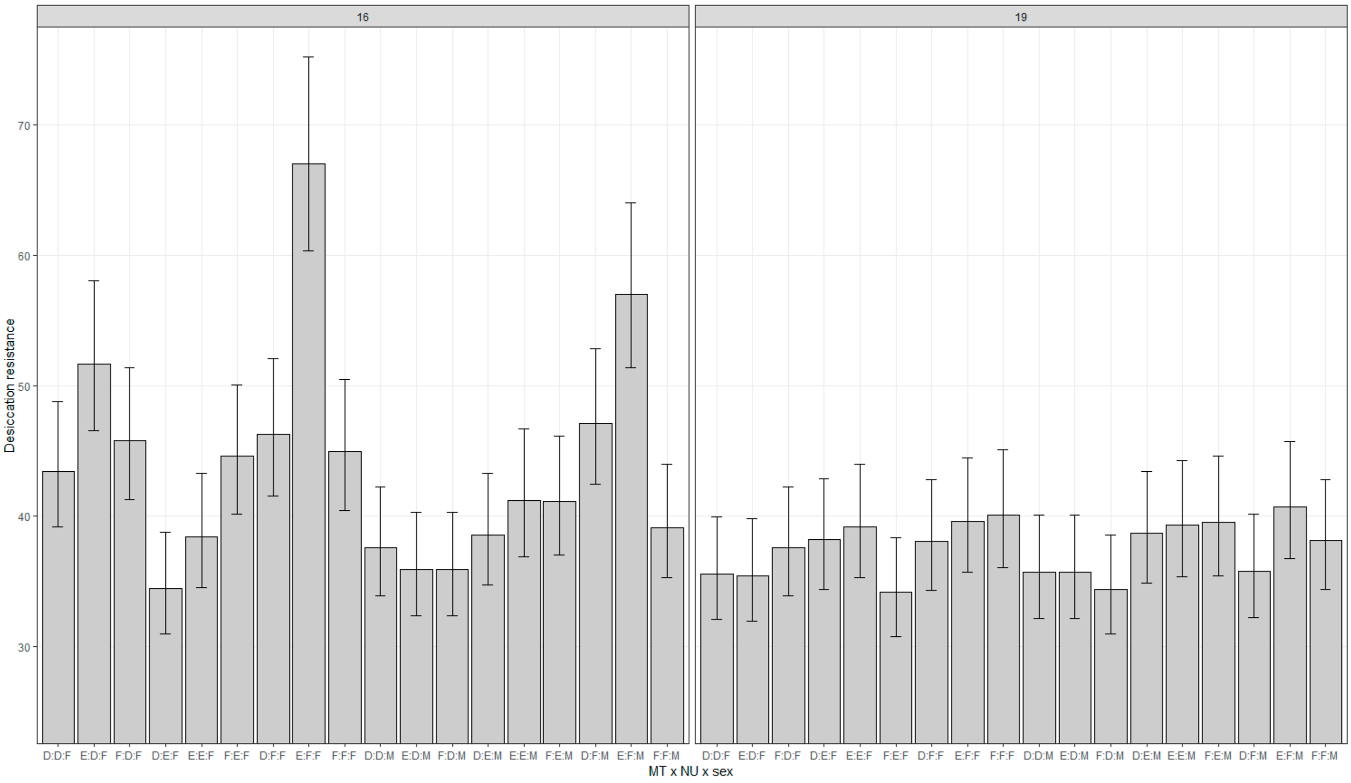

Supplementary Figure S2. Mean survival times in hours for the desiccation experiment for all combinations of genotypes and sex from the EB II on two experimental temperatures. D, E and F denote MNILs (the first letter marks mtDNA haplotype (MT), the second letter marks nuDNA background (NU)), F and M denote females and males, respectively (SEX). Error bars represent 95% confidence intervals.

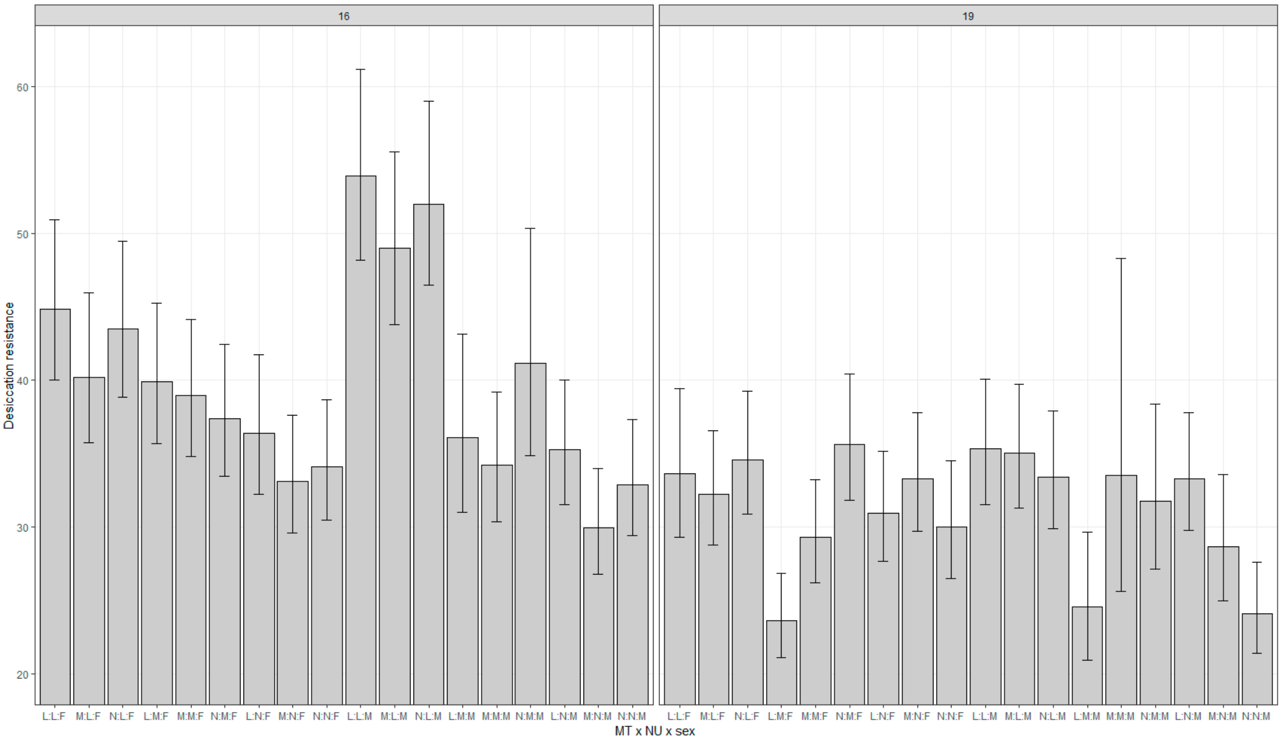

Supplementary Figure S3. Mean survival times in hours for the desiccation experiment for all combinations of genotypes and sex from the EB III on two experimental temperatures. L, M and N denote MNILs (the first letter marks mtDNA haplotype (MT), the second letter marks nuDNA background (NU)), F and M denote females and males, respectively (SEX). Error bars represent 95% confidence intervals.

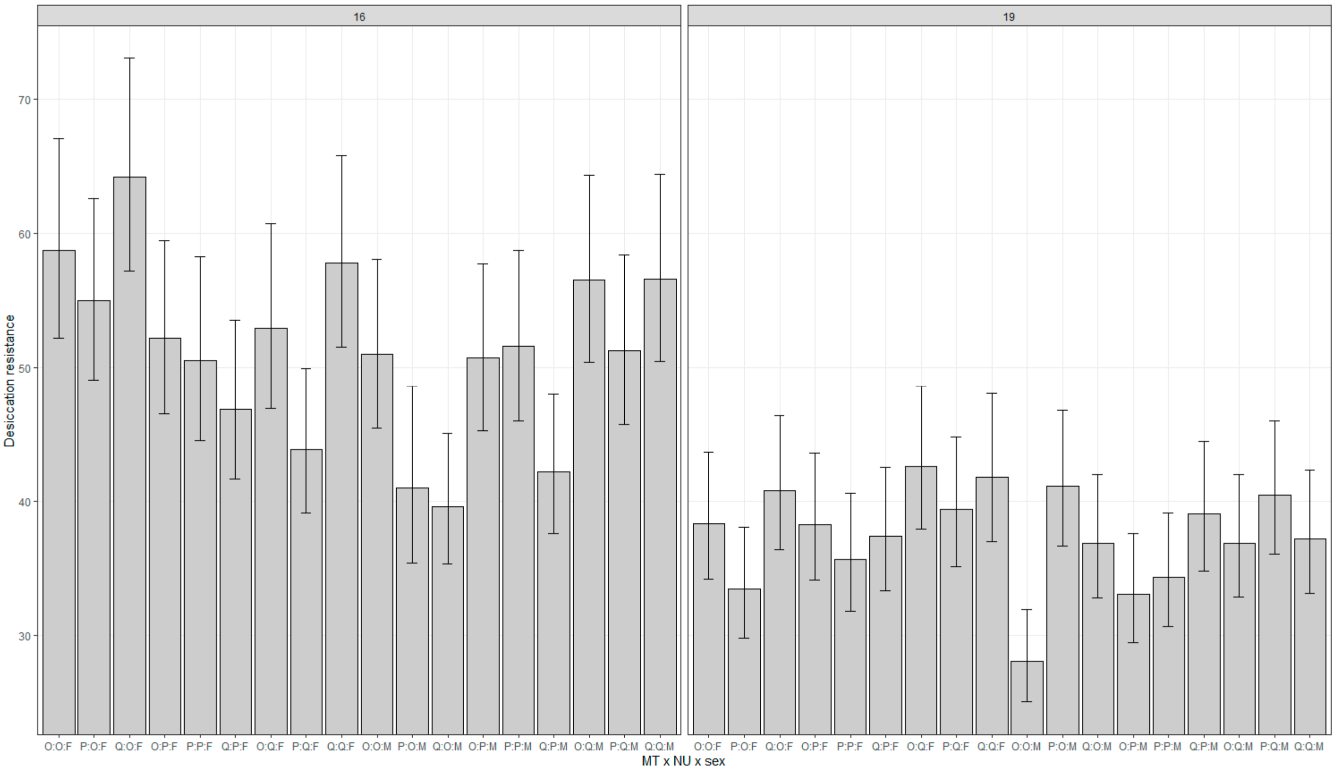

Supplementary Figure S4. Mean survival times in hours for the desiccation experiment for all combinations of genotypes and sex from the EB IV on two experimental temperatures. O, P and Q denote MNILs (the first letter marks mtDNA haplotype (MT), the second letter marks nuDNA background (NU)), F and M denote females and males, respectively (SEX). Error bars represent 95% confidence intervals.

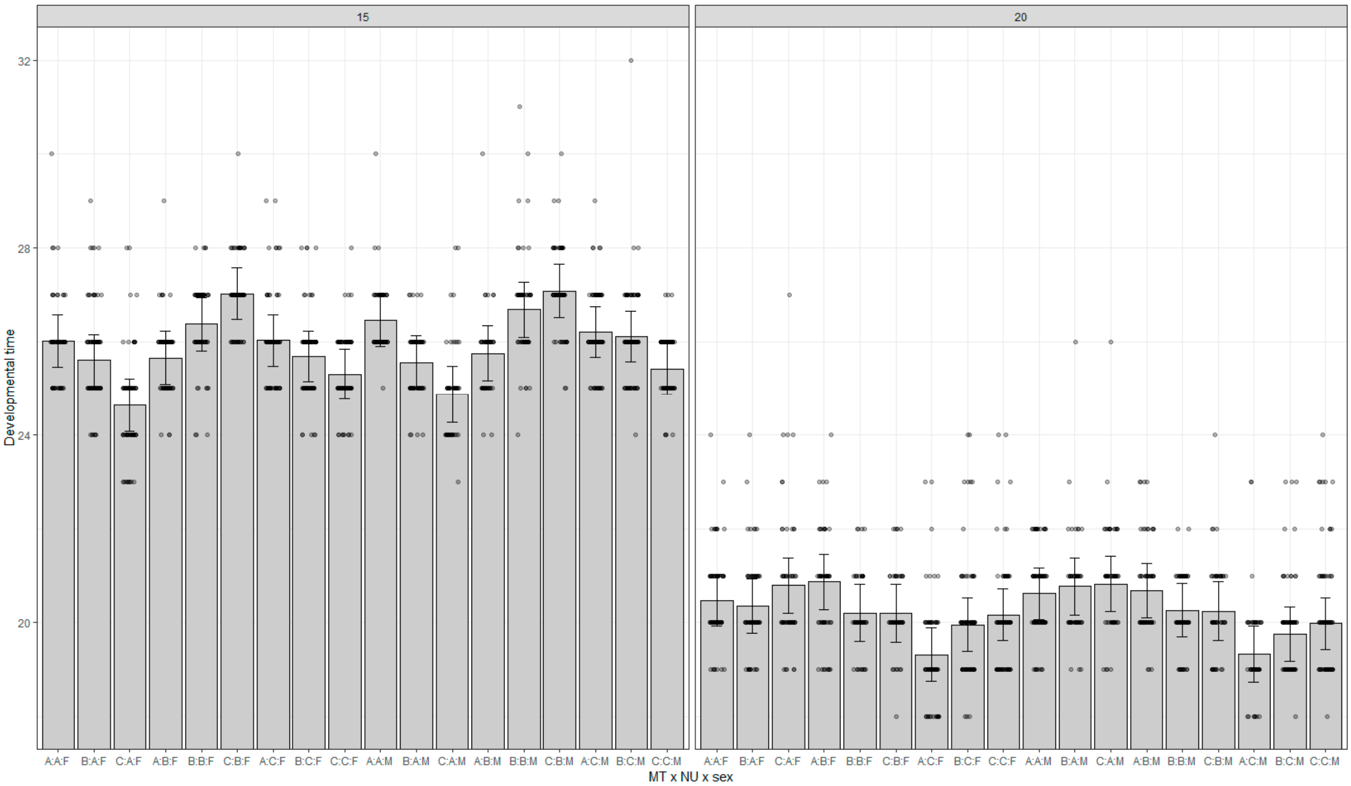

Supplementary Figure S5. Mean developmental times in days for all combinations of genotypes and sex from the EB I on two experimental temperatures. A, B and C denote MNILs (the first letter marks mtDNA haplotype (MT), the second letter marks nuDNA background (NU)), F and M denote females and males, respectively (SEX). Error bars represent 95% confidence intervals.

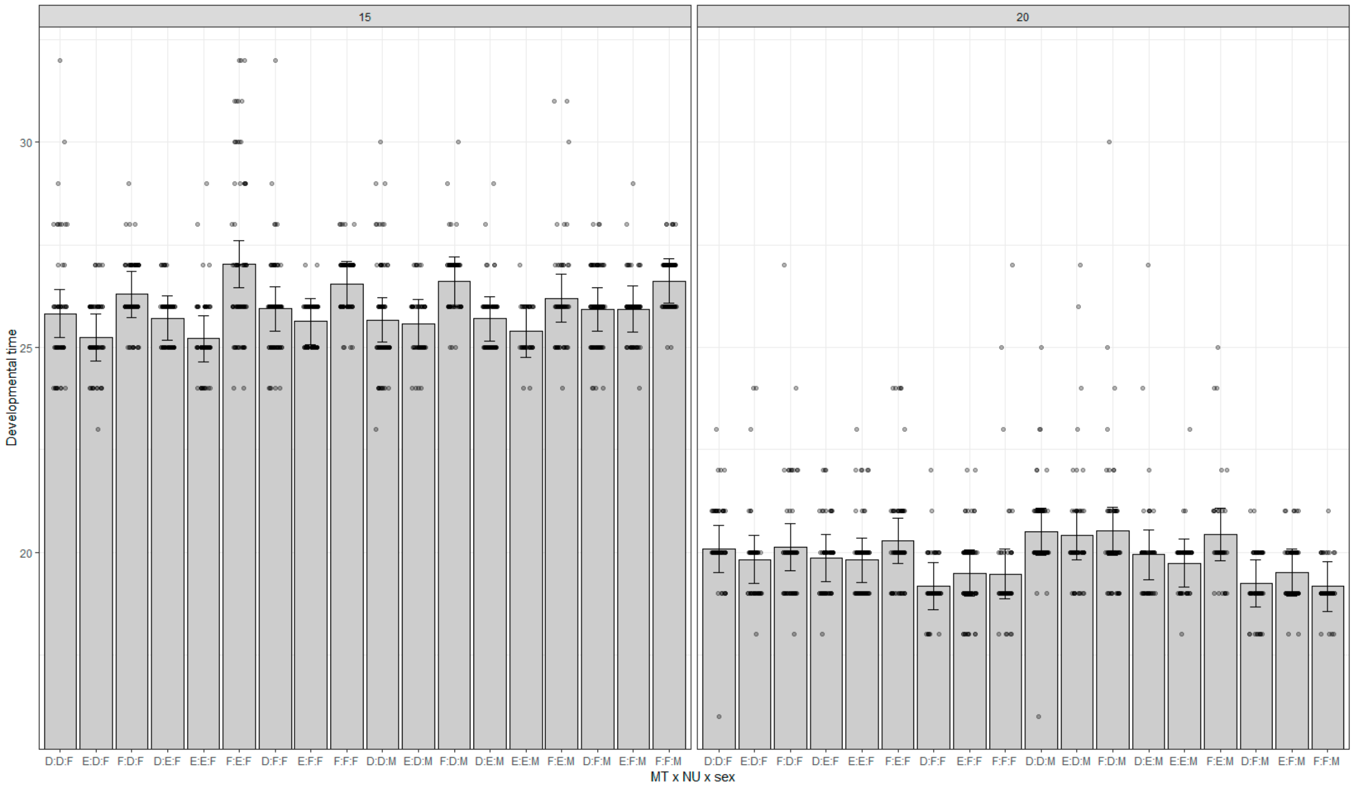

Supplementary Figure S6. Mean developmental times in days for all combinations of genotypes and sex from the EB II on two experimental temperatures. D, E and F denote MNILs (the first letter marks mtDNA haplotype (MT), the second letter marks nuDNA background (NU)), F and M denote females and males, respectively (SEX). Error bars represent 95% confidence intervals.

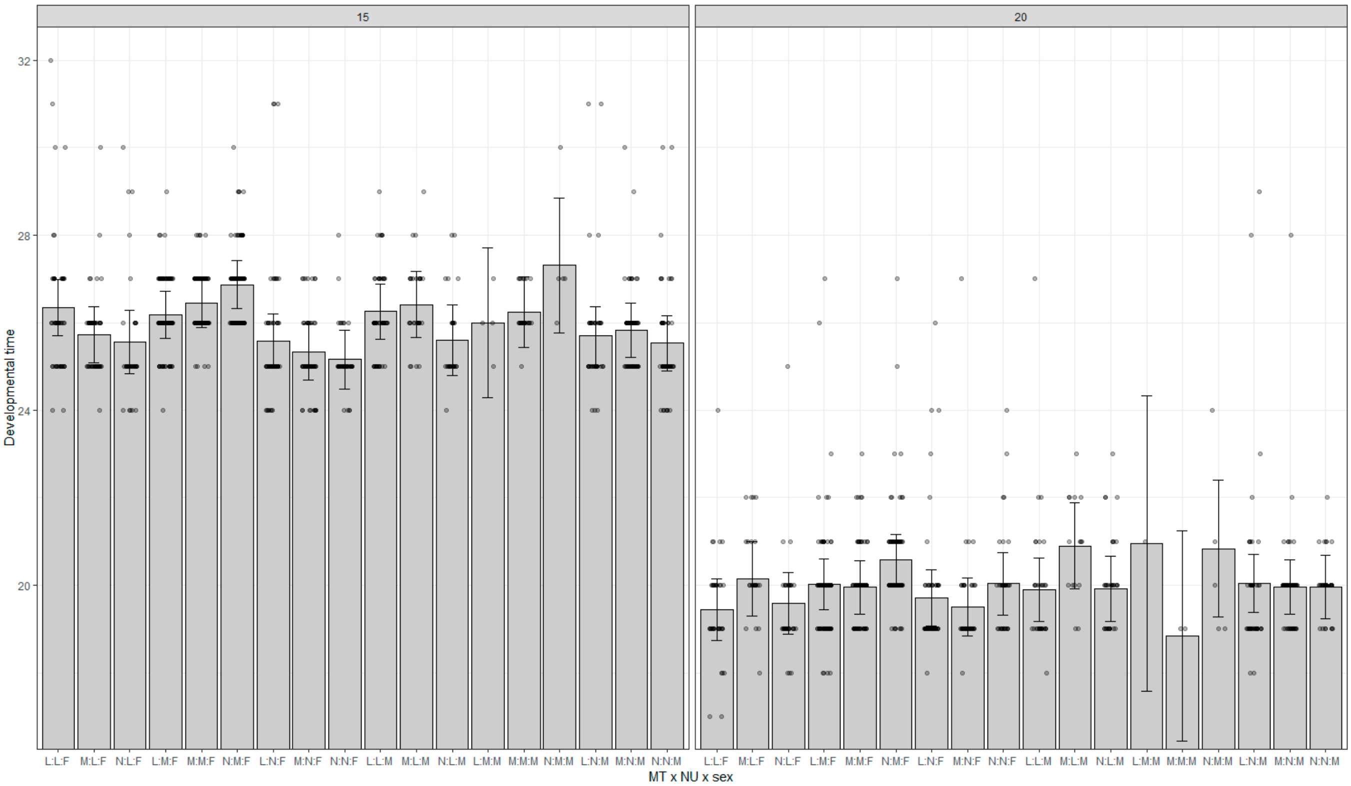

Supplementary Figure S7. Mean developmental times in days for all combinations of genotypes and sex from the EB III on two experimental temperatures. L, M and N denote MNILs (the first letter marks mtDNA haplotype (MT), the second letter marks nuDNA background (NU)), F and M denote females and males, respectively (SEX). Error bars represent 95% confidence intervals.

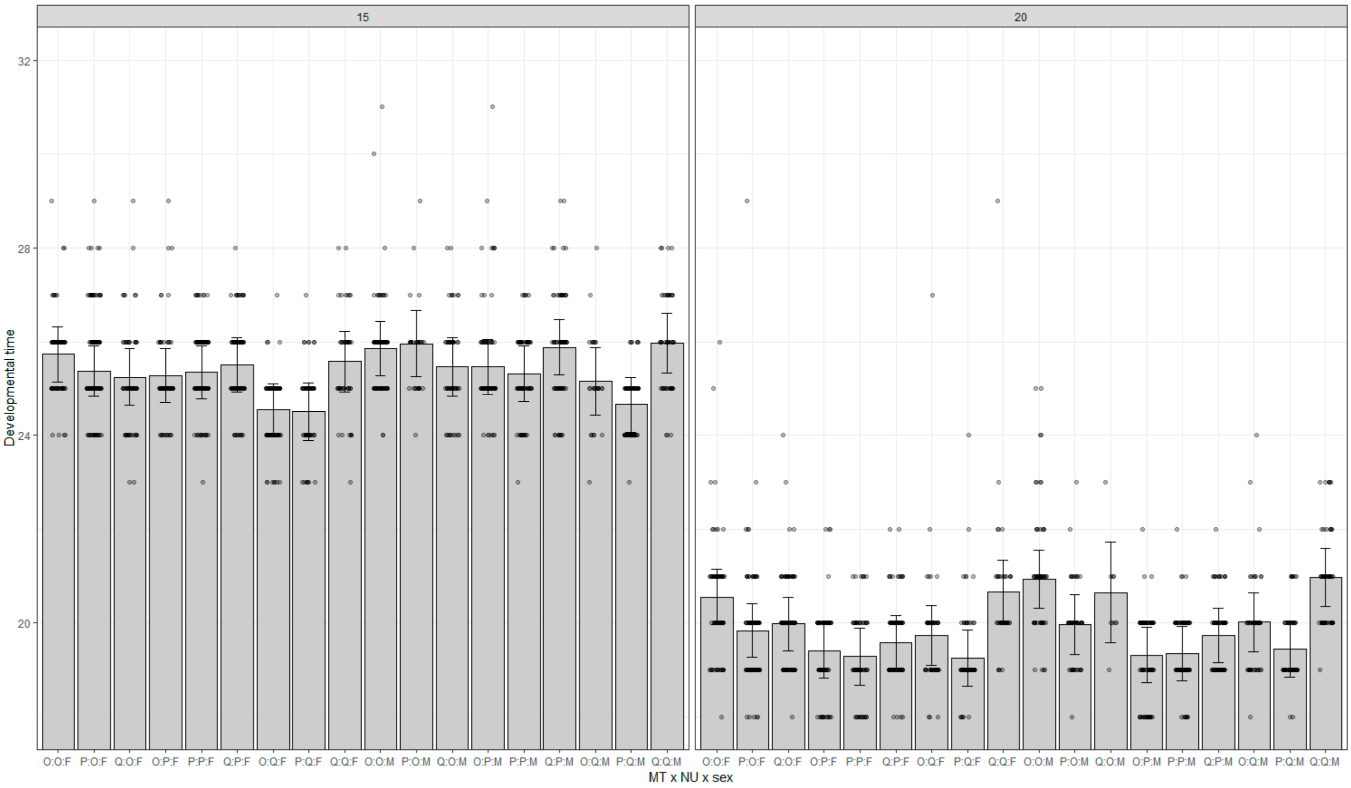

Supplementary Figure S8. Mean developmental times in days for all combinations of genotypes and sex from the EB IV on two experimental temperatures. O, P and Q denote MNILs (the first letter marks mtDNA haplotype (MT), the second letter marks nuDNA background (NU)), F and M denote females and males, respectively (SEX). Error bars represent 95% confidence intervals.

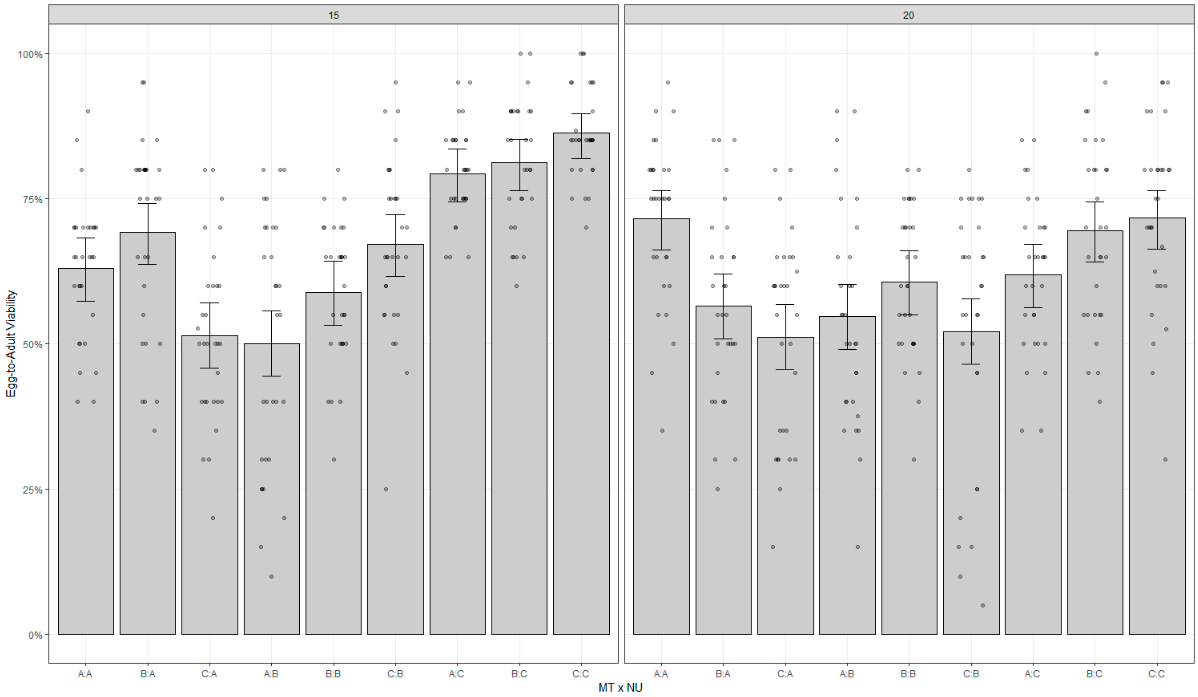

Supplementary Figure S9. Mean egg-to-adult viability scores for all combinations of genotypes from the EB I on two experimental temperatures. A, B and C denote MNILs (the first letter marks mtDNA haplotype (MT), the second letter marks nuDNA background (NU)). Error bars represent 95% confidence intervals.

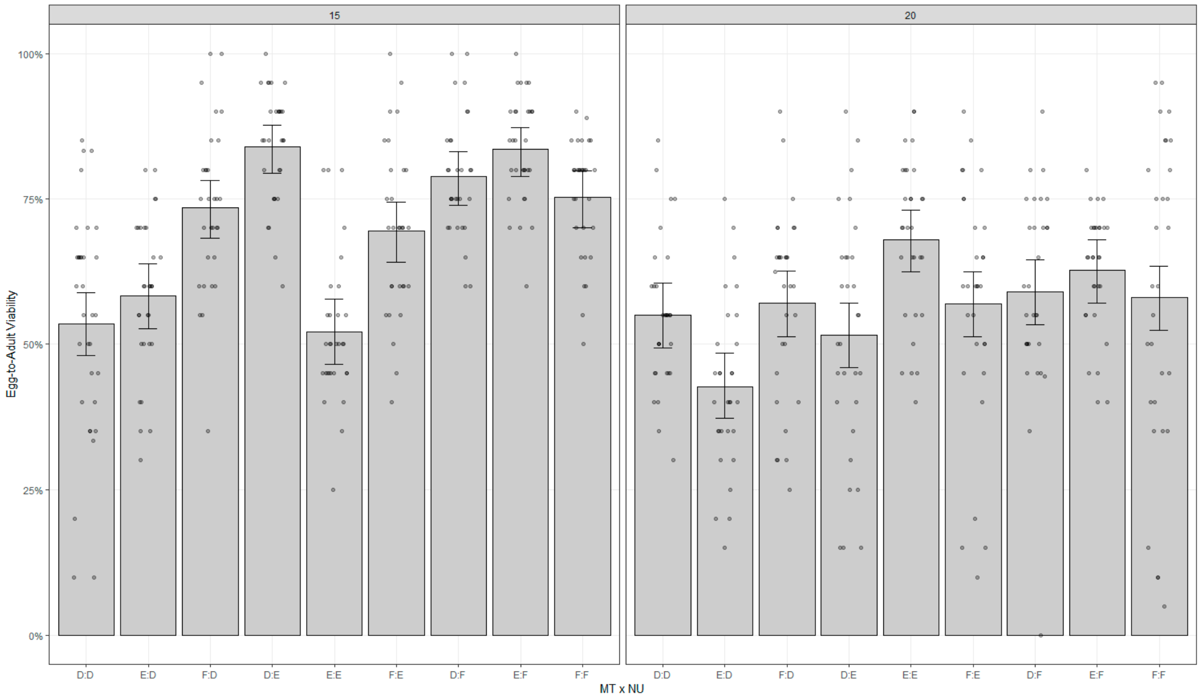

Supplementary Figure S10. Mean egg-to-adult viability scores for all combinations of genotypes from the EB II on two experimental temperatures. D, E and F denote MNILs (the first letter marks mtDNA haplotype (MT), the second letter marks nuDNA background (NU)). Error bars represent 95% confidence intervals.

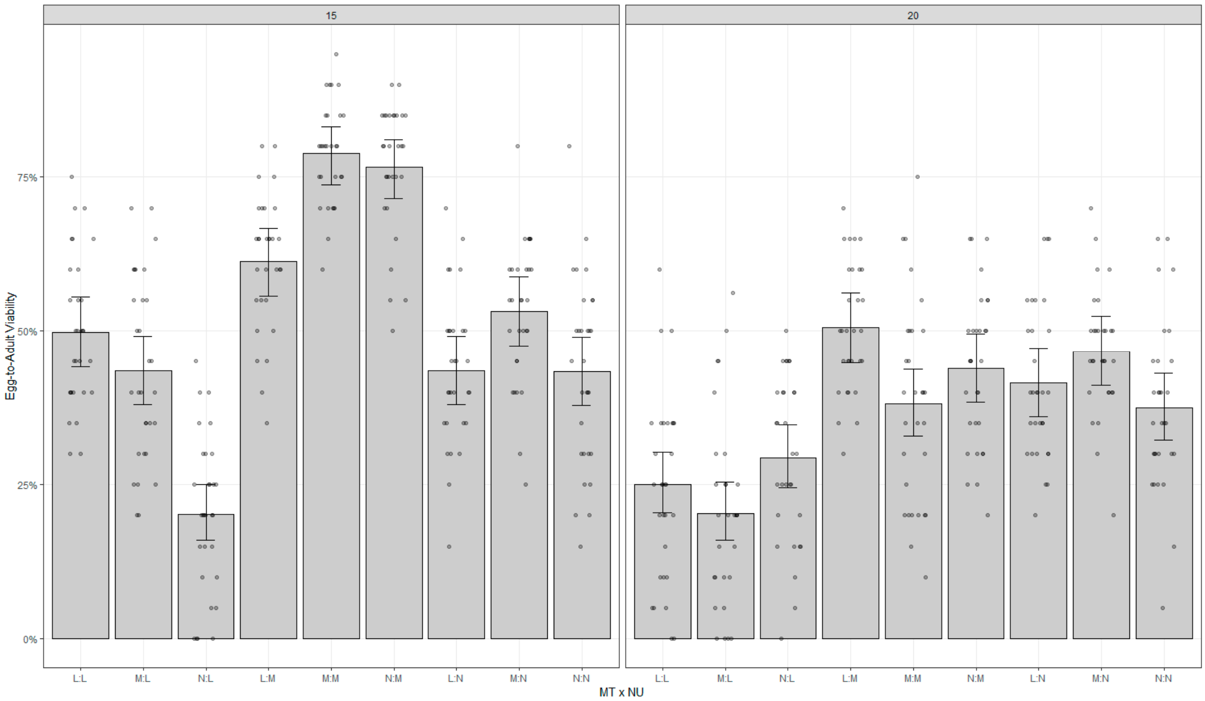

Supplementary Figure S11. Mean egg-to-adult viability scores for all combinations of genotypes from the EB III on two experimental temperatures. L, M and N denote MNILs (the first letter marks mtDNA haplotype (MT), the second letter marks nuDNA background (NU)). Error bars represent 95% confidence intervals.

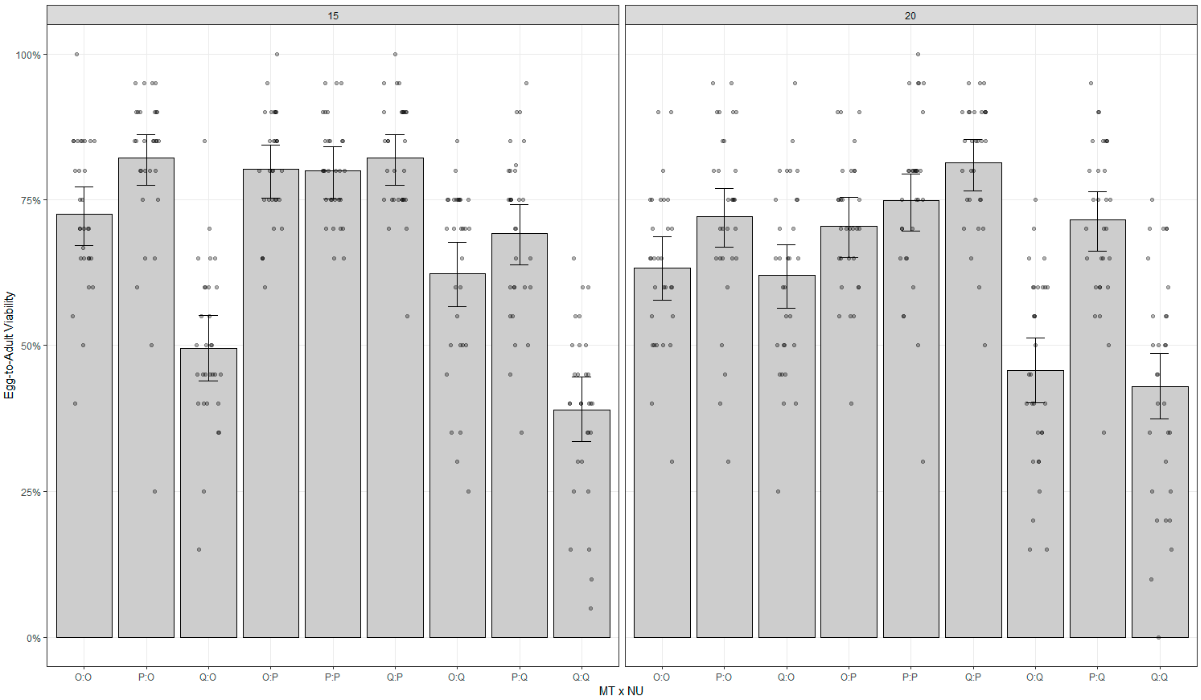

Supplementary Figure S12. Mean egg-to-adult viability scores for all combinations of genotypes from the EB IV on two experimental temperatures. O, P and Q denote MNILs (the first letter marks mtDNA haplotype (MT), the second letter marks nuDNA background (NU)). Error bars represent 95% confidence intervals.

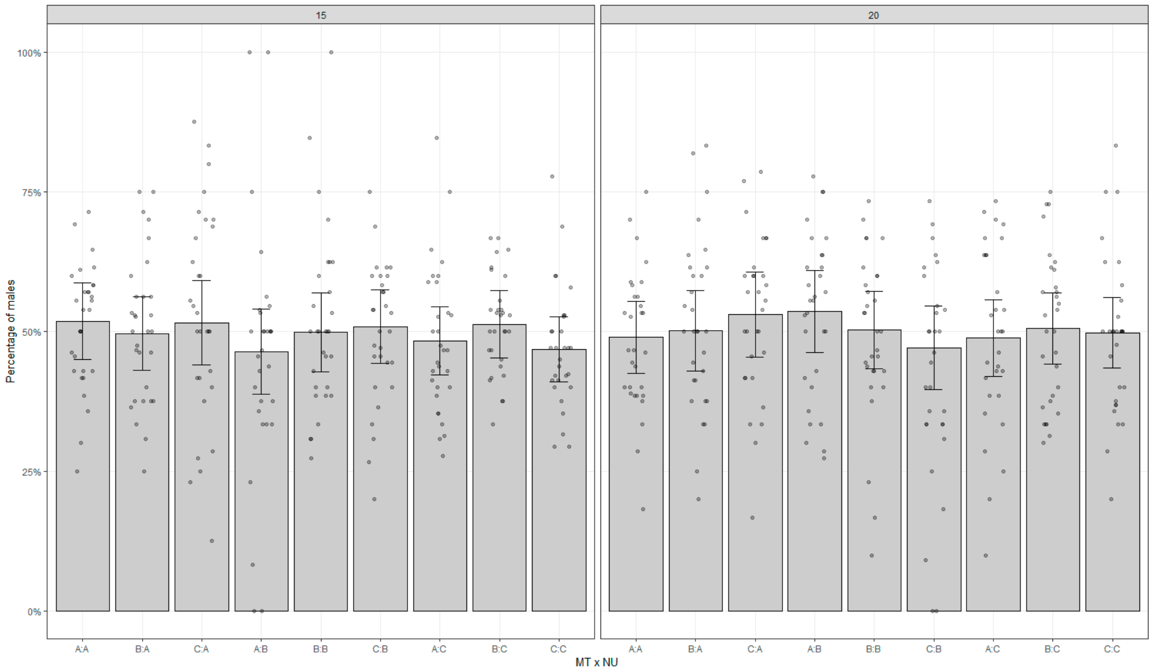

Supplementary Figure S13. Mean proportion of males for all combinations of genotypes from EB I on two experimental temperatures. A, B and C denote MNILs (the first letter marks mtDNA haplotype (MT), the second letter marks nuDNA background (NU)). Error bars represent 95% confidence intervals.

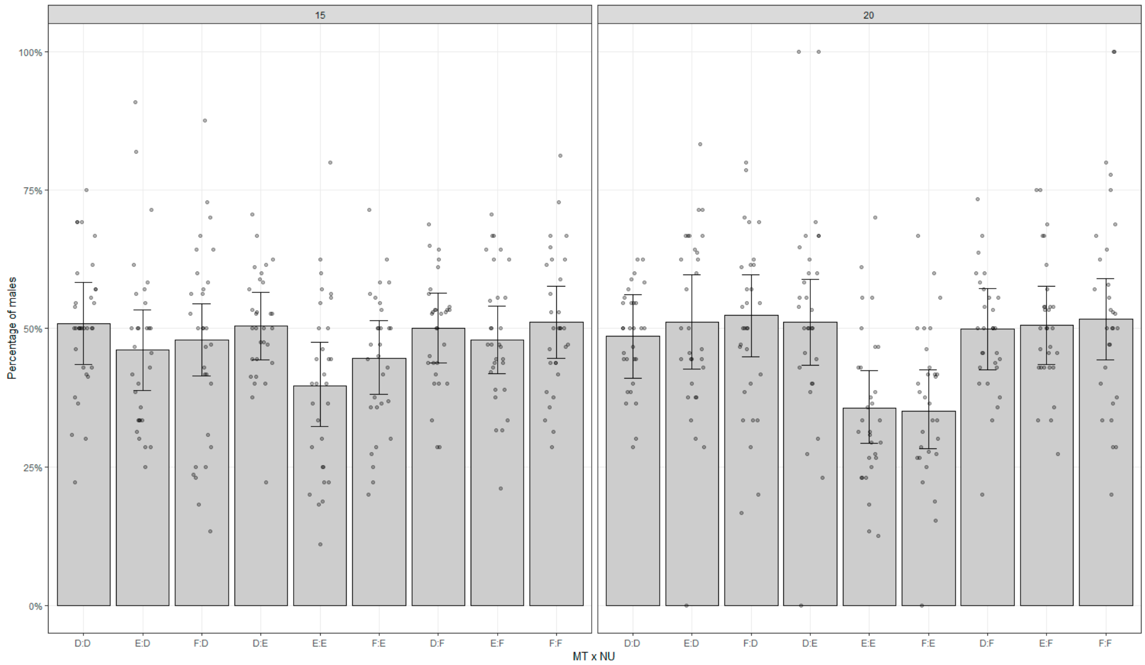

Supplementary Figure S14. Mean proportion of males for all combinations of genotypes from EB II on two experimental temperatures. D, E and F denote MNILs (the first letter marks mtDNA haplotype (MT), the second letter marks nuDNA background (NU)). Error bars represent 95% confidence intervals.

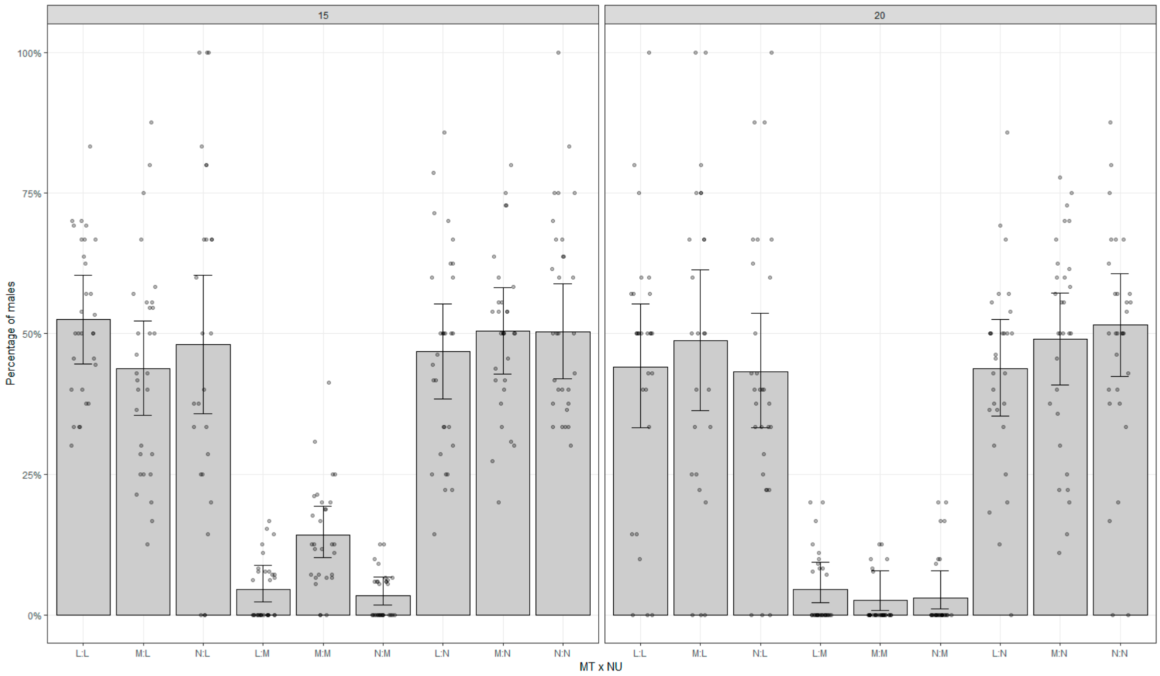

Supplementary Figure S15. Mean proportion of males for all combinations of genotypes from EB III on two experimental temperatures. L, M and N denote MNILs (the first letter marks mtDNA haplotype (MT), the second letter marks nuDNA background (NU)). Error bars represent 95% confidence intervals.

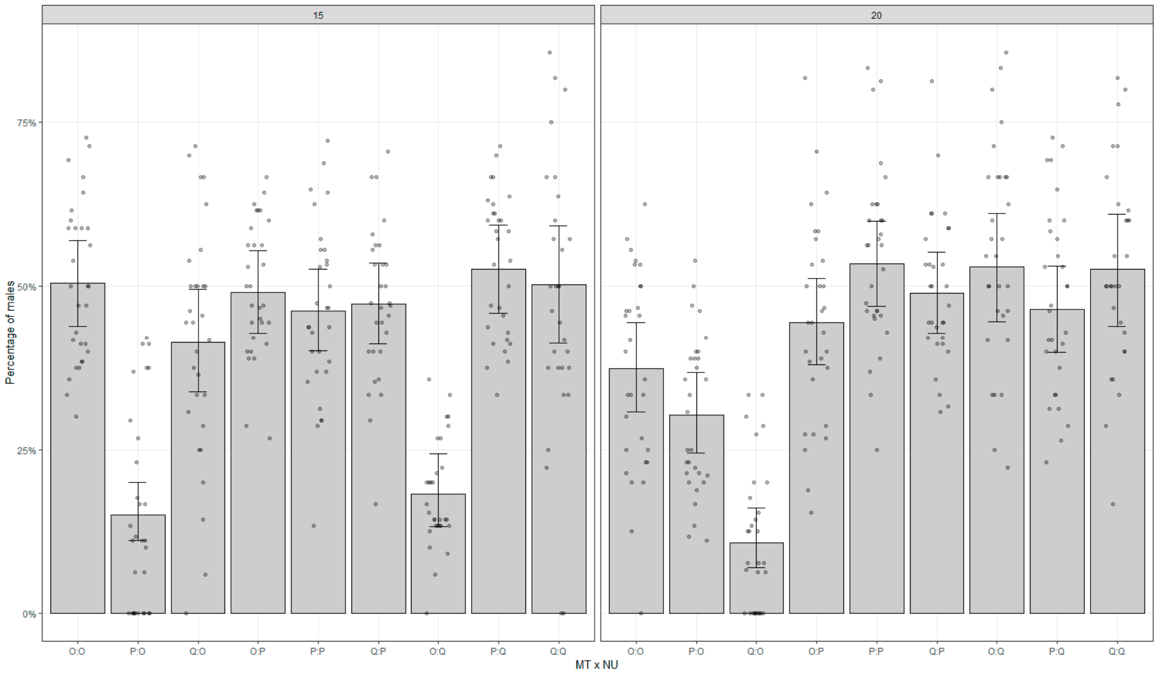

Supplementary Figure S16. Mean proportion of males for all combinations of genotypes from EB IV on two experimental temperatures. O, P and Q denote MNILs (the first letter marks mtDNA haplotype (MT), the second letter marks nuDNA background (NU)). Error bars represent 95% confidence intervals.
