# Supplementary figures and images for: Temperature-Specific and Sex-Specific Fitness Effects of Sympatric Mitochondrial and Mito-Nuclear Variation in Drosophila obscura
Source: Insects. 2022 Jan 28;13(2):139. doi: 10.3390/insects13020139 (PMC8880146; doi:10.3390/insects13020139)

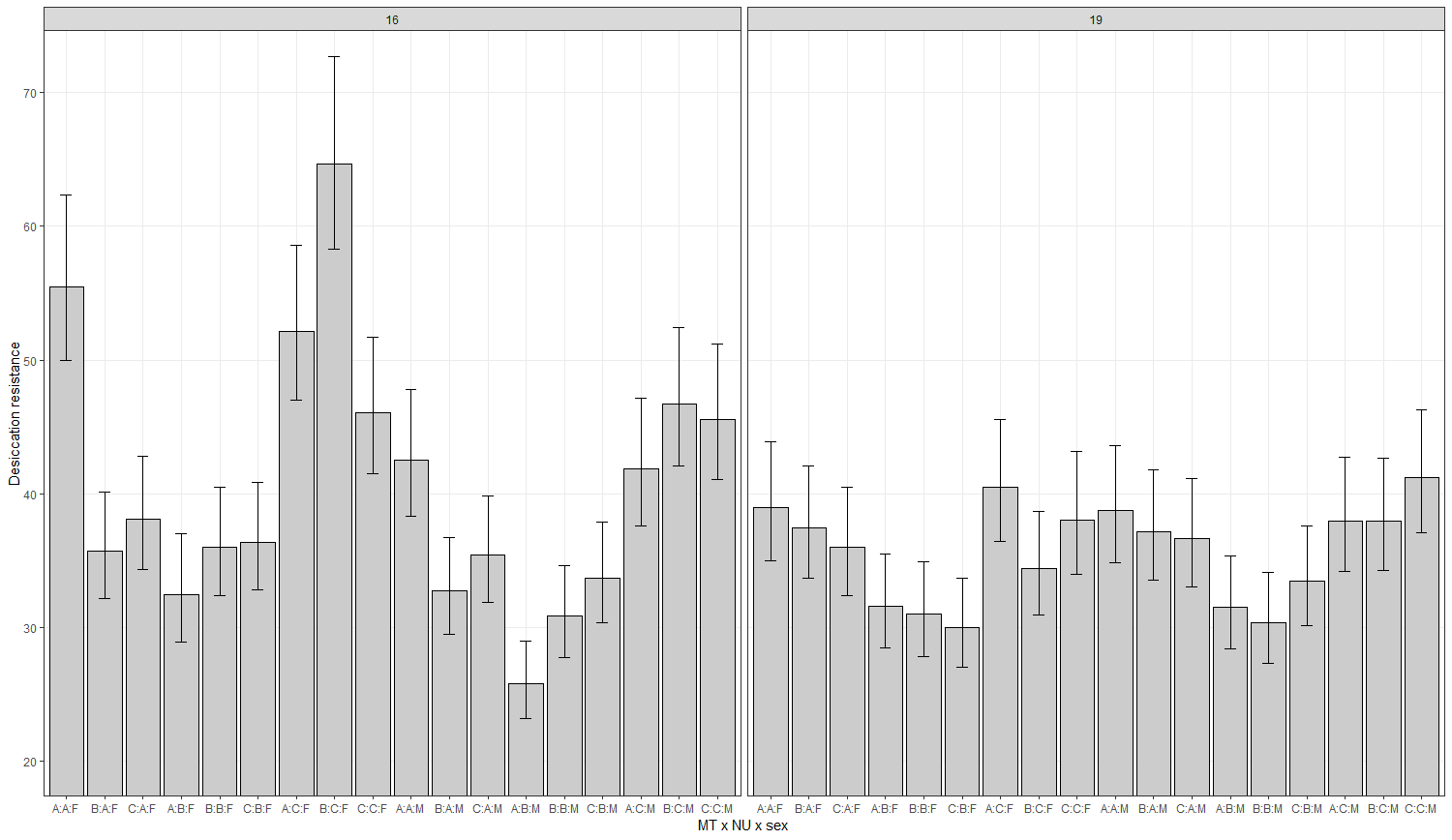

Supplement: Supplementary file 1 [file insects-13-00139-s001.zip › Supplementary Figure 1.png]

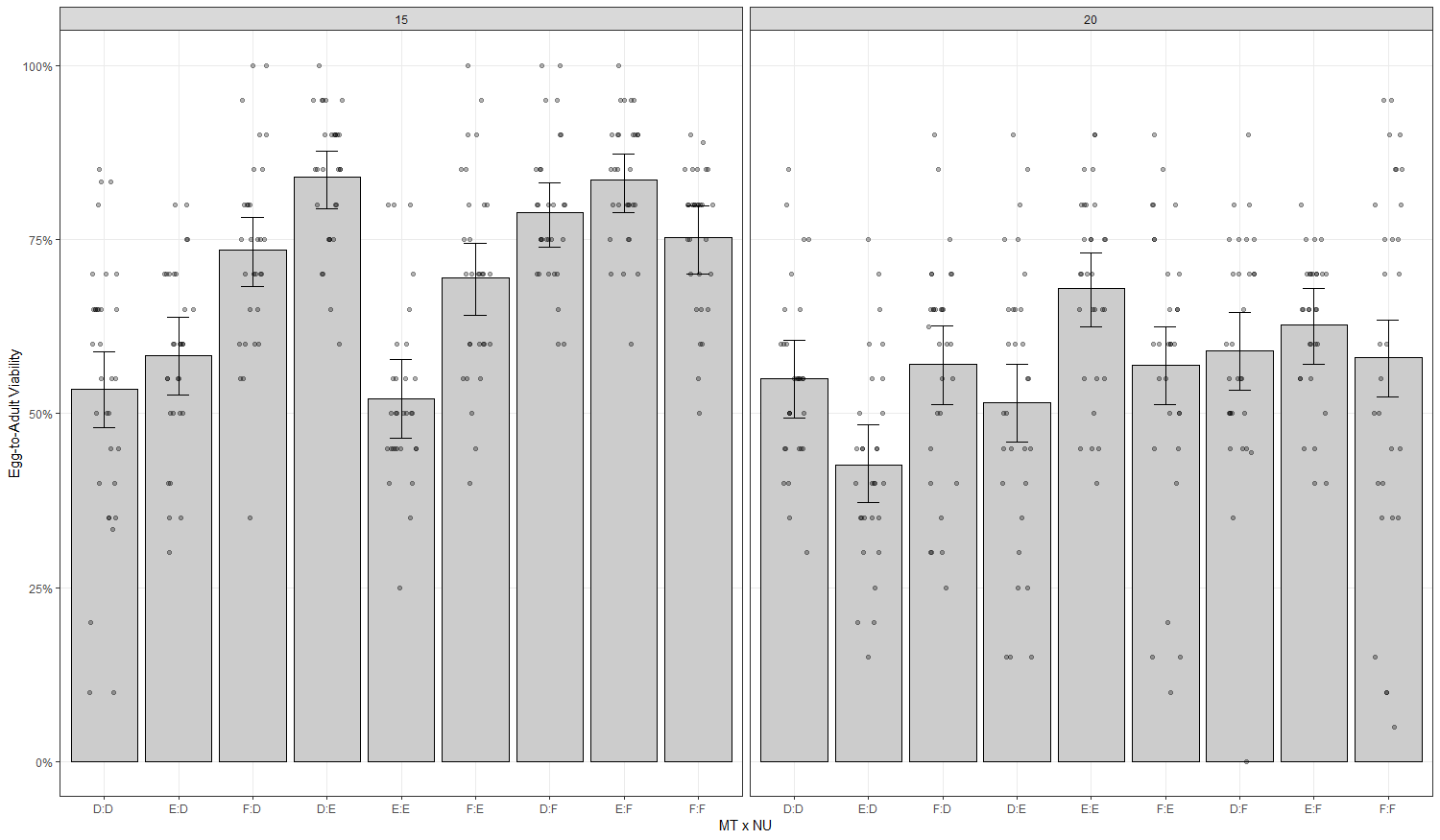

Supplement: Supplementary file 1 [file insects-13-00139-s001.zip › Supplementary Figure 10.png]

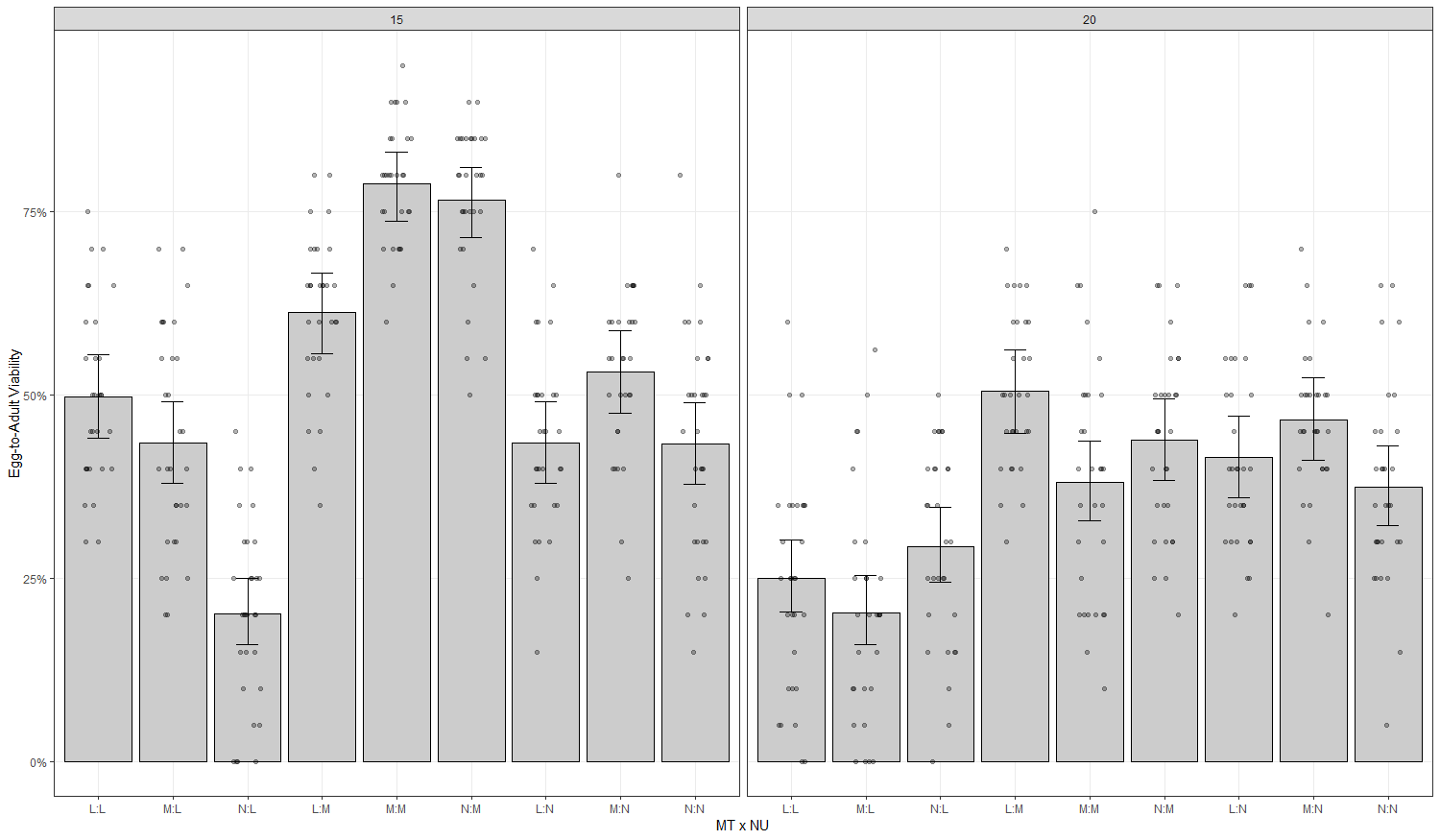

Supplement: Supplementary file 1 [file insects-13-00139-s001.zip › Supplementary Figure 11.png]

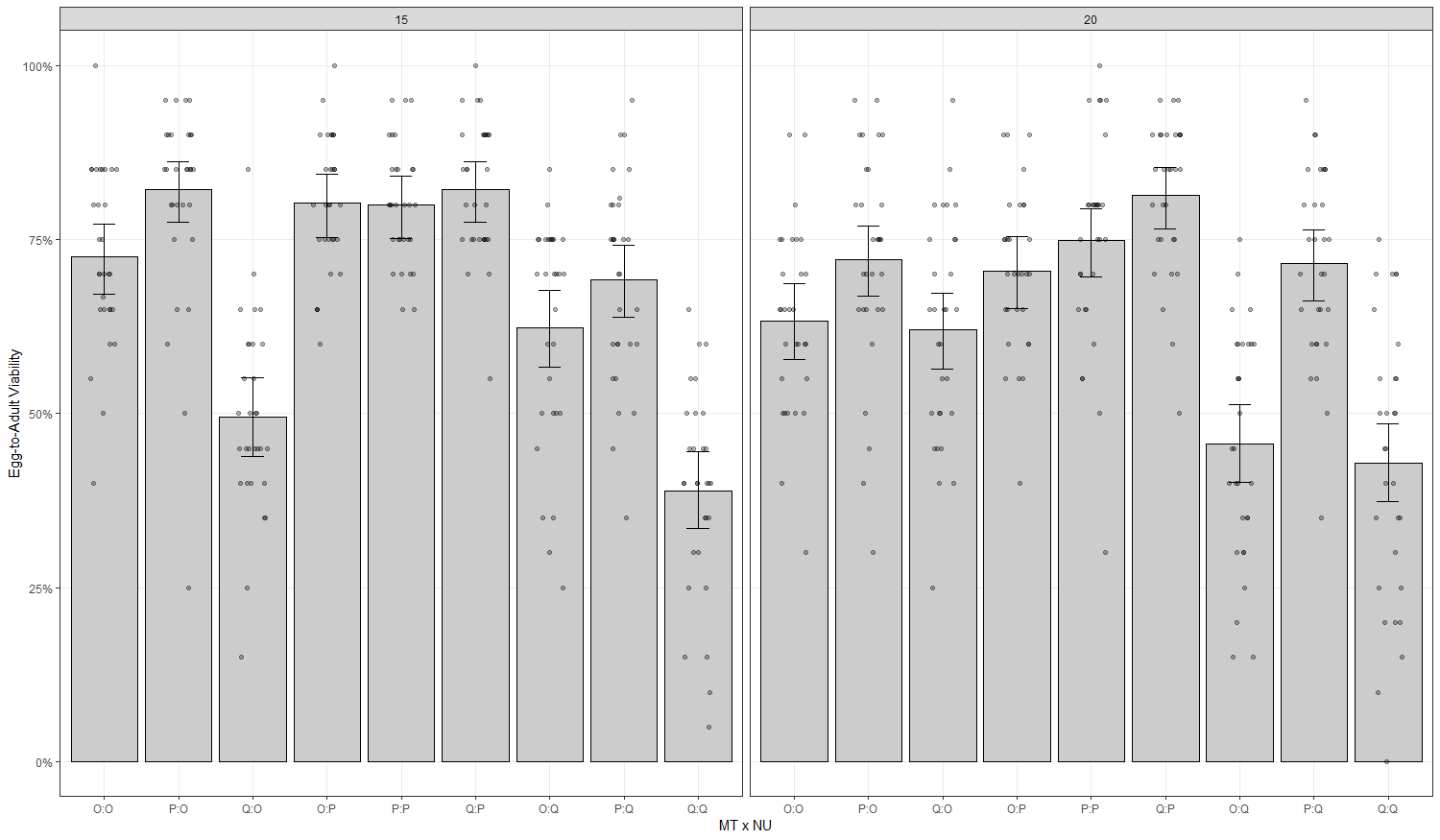

Supplement: Supplementary file 1 [file insects-13-00139-s001.zip › Supplementary Figure 12.png]

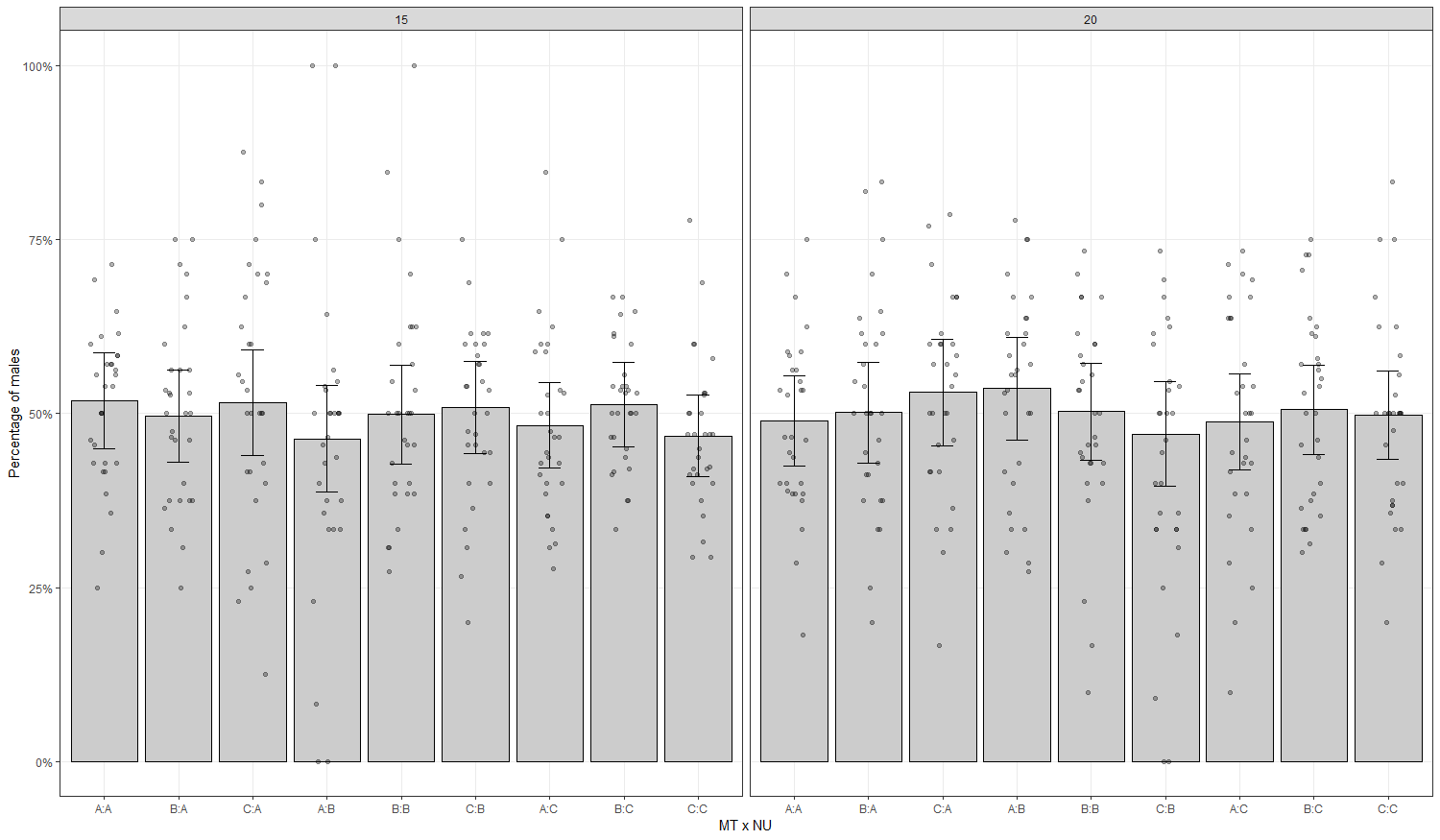

Supplement: Supplementary file 1 [file insects-13-00139-s001.zip › Supplementary Figure 13.png]

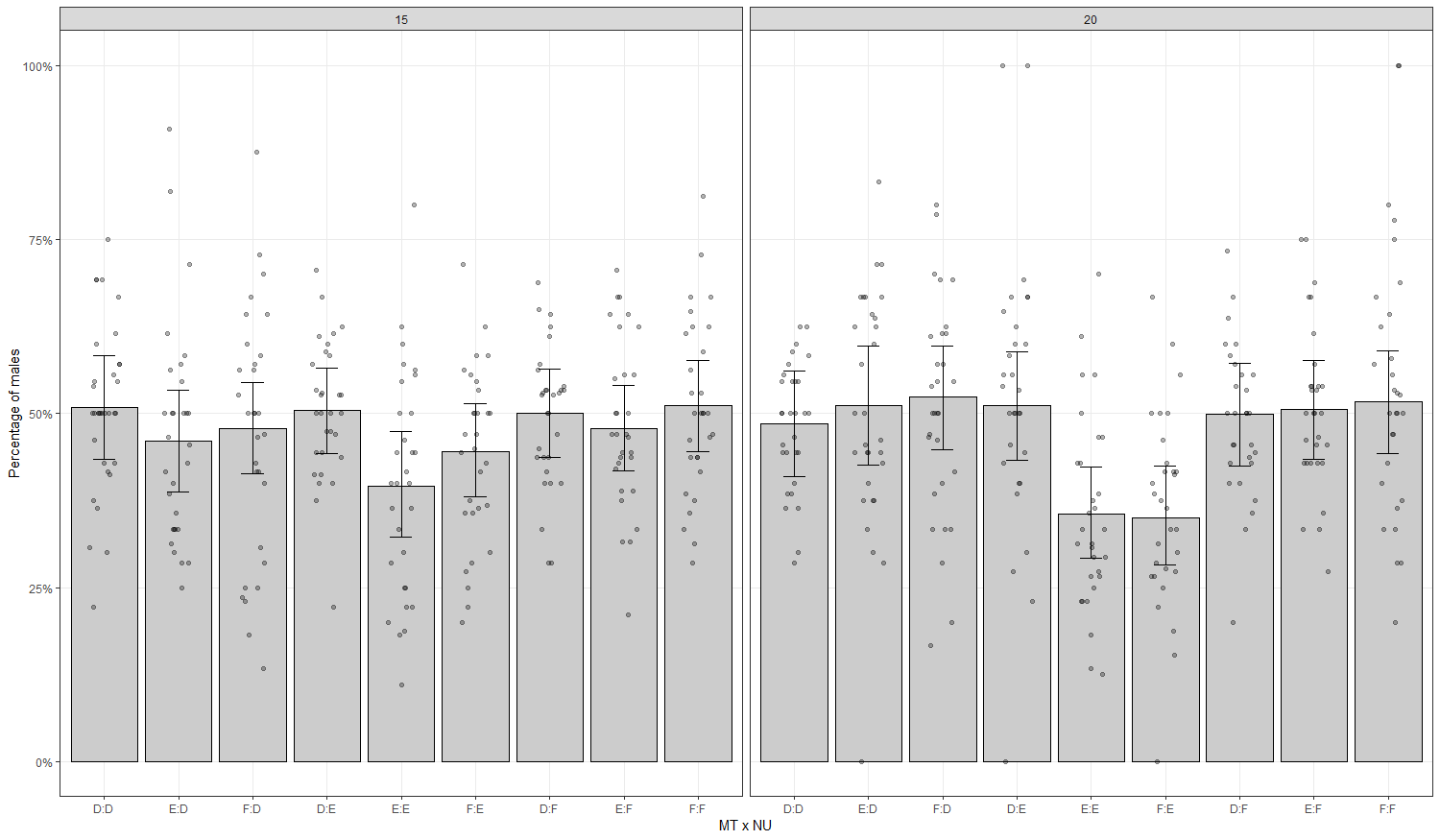

Supplement: Supplementary file 1 [file insects-13-00139-s001.zip › Supplementary Figure 14.png]

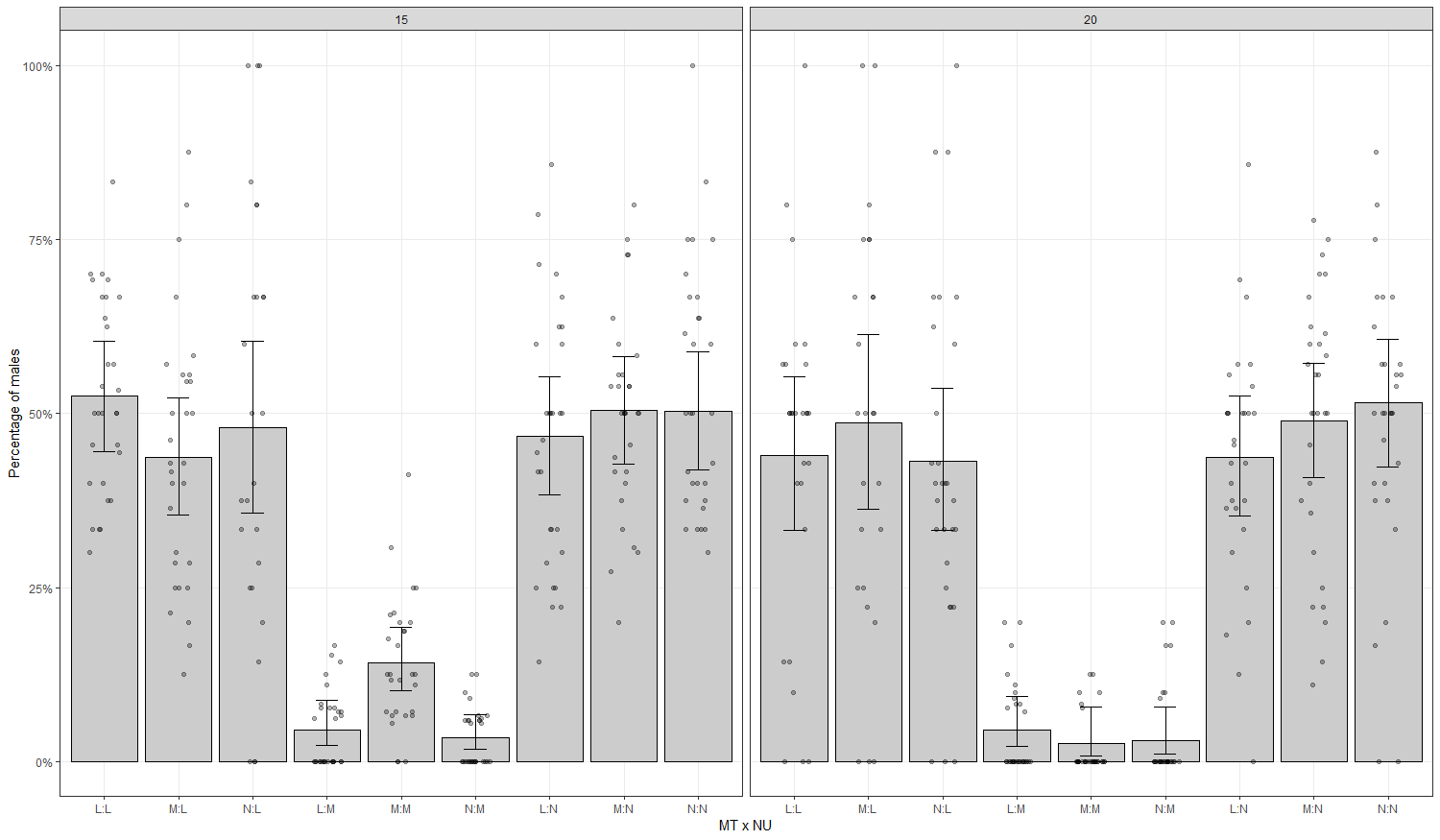

Supplement: Supplementary file 1 [file insects-13-00139-s001.zip › Supplementary Figure 15.png]

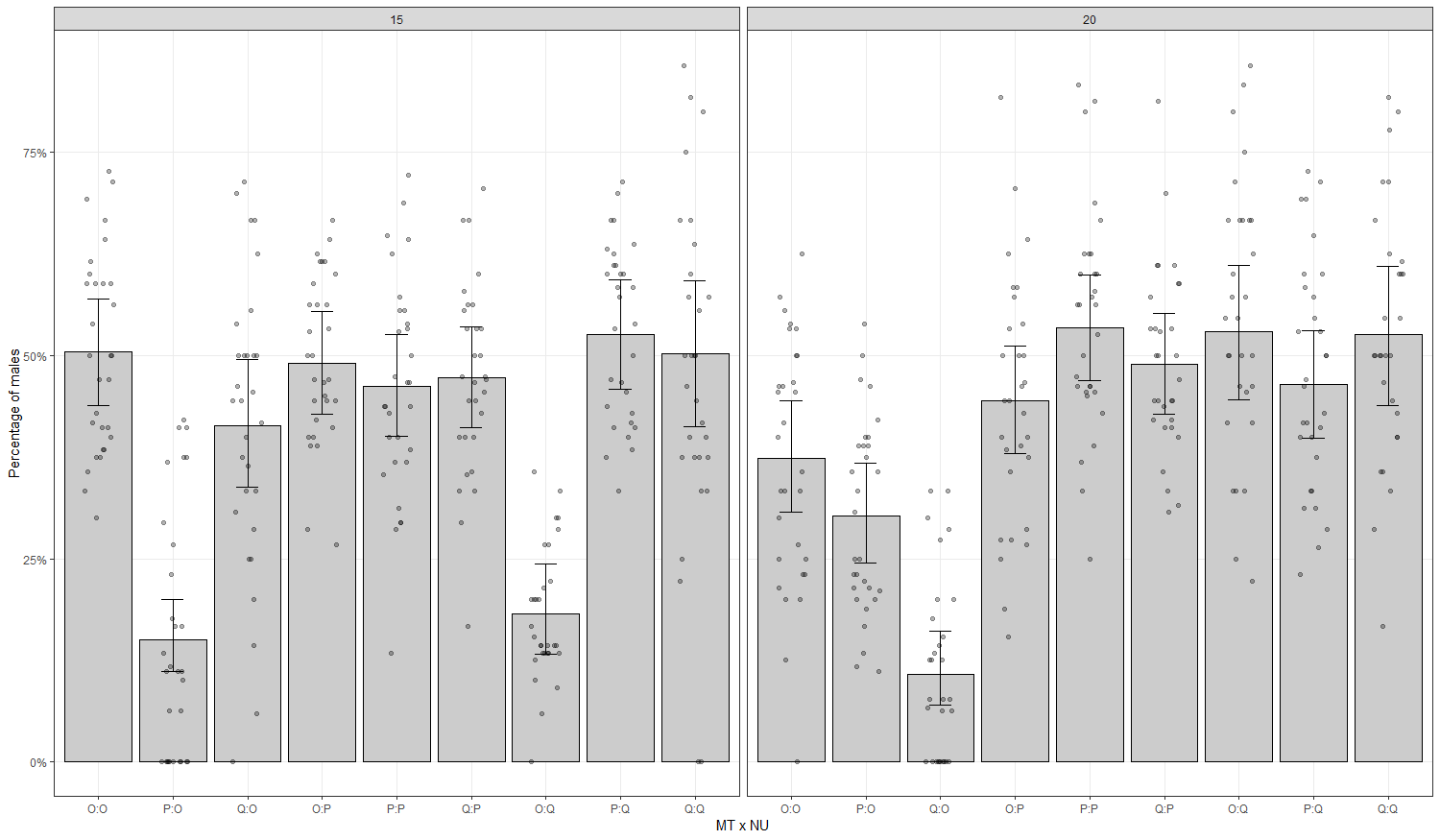

Supplement: Supplementary file 1 [file insects-13-00139-s001.zip › Supplementary Figure 16.png]

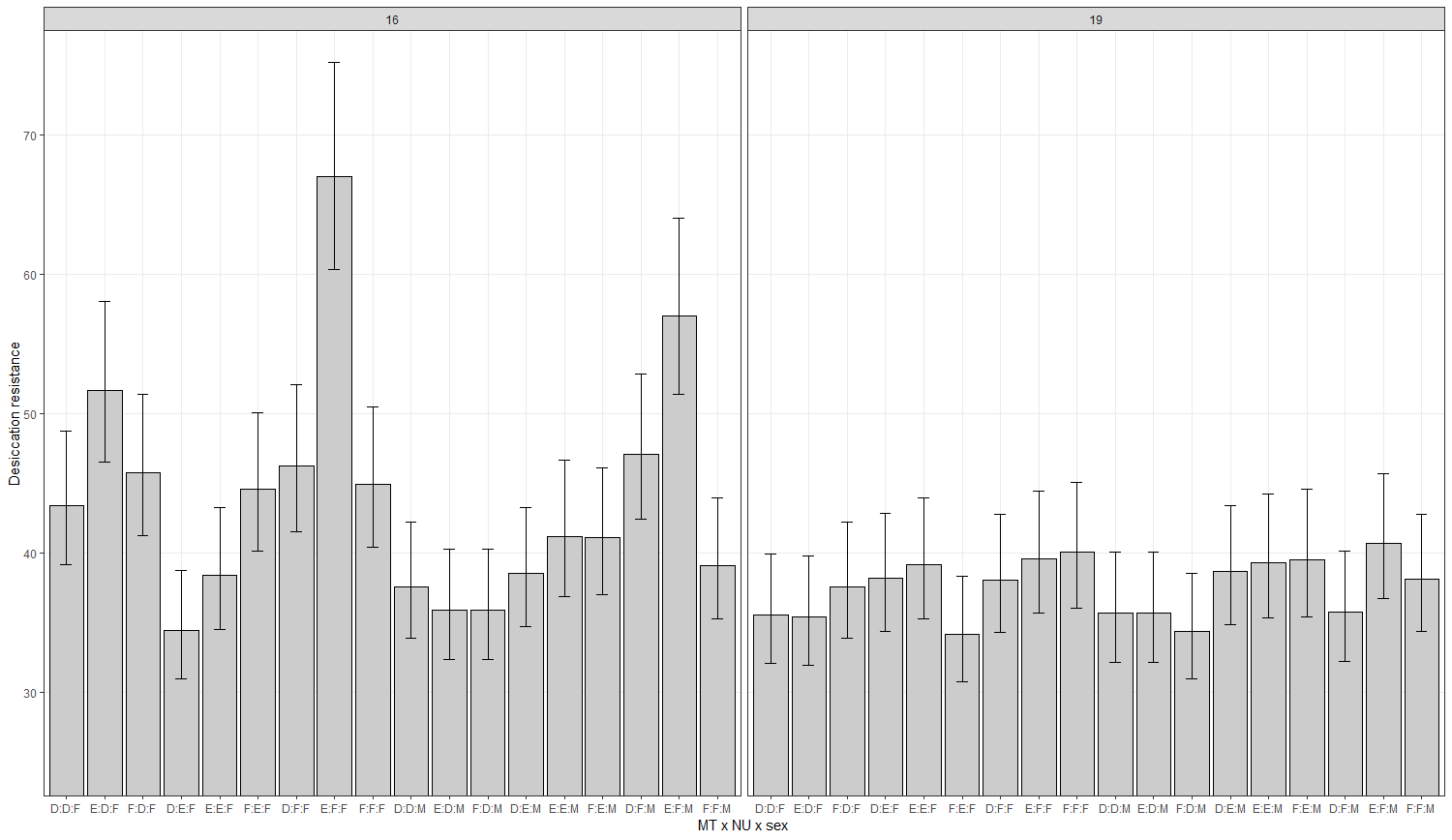

Supplement: Supplementary file 1 [file insects-13-00139-s001.zip › Supplementary Figure 2.png]

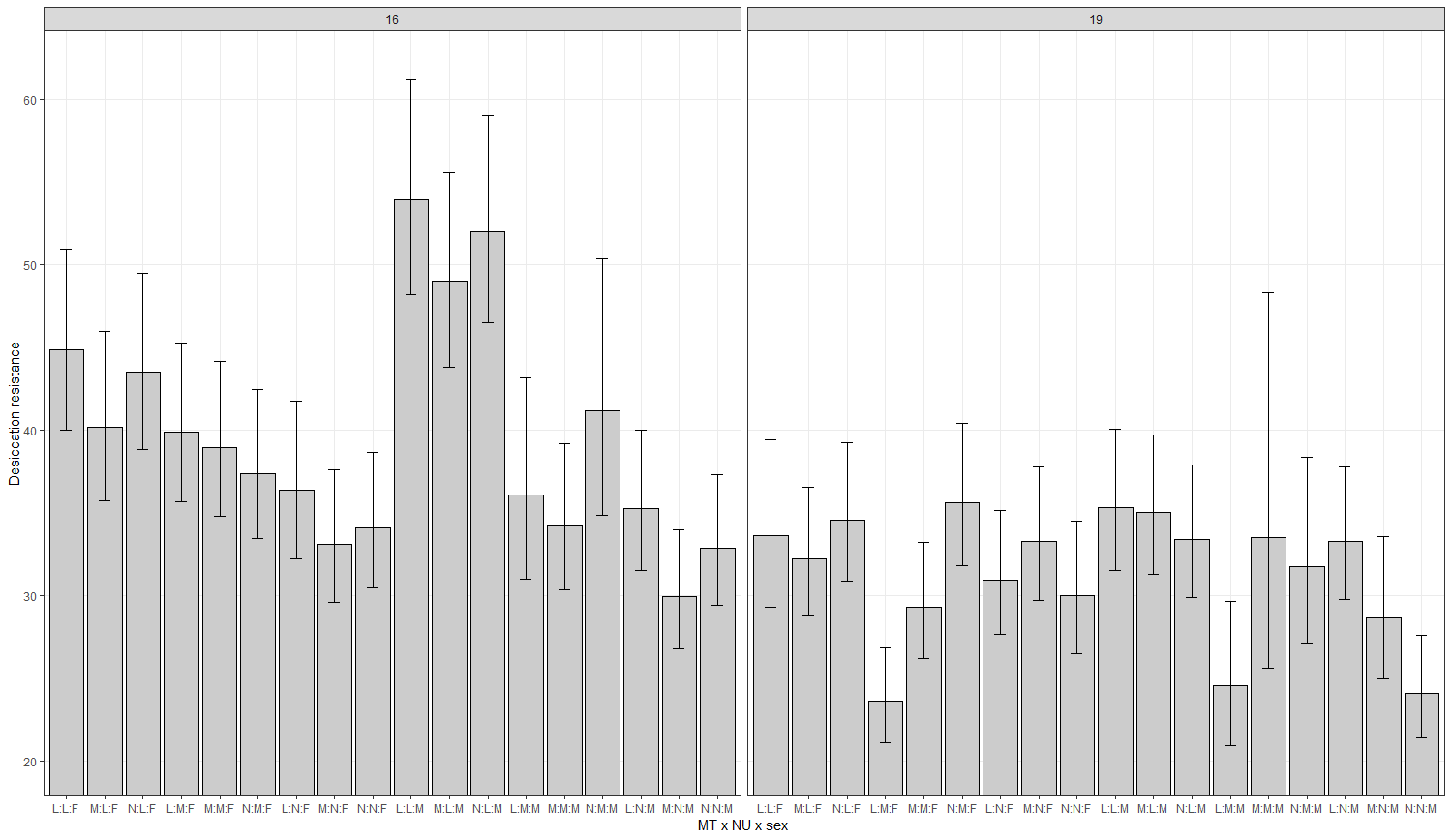

Supplement: Supplementary file 1 [file insects-13-00139-s001.zip › Supplementary Figure 3.png]

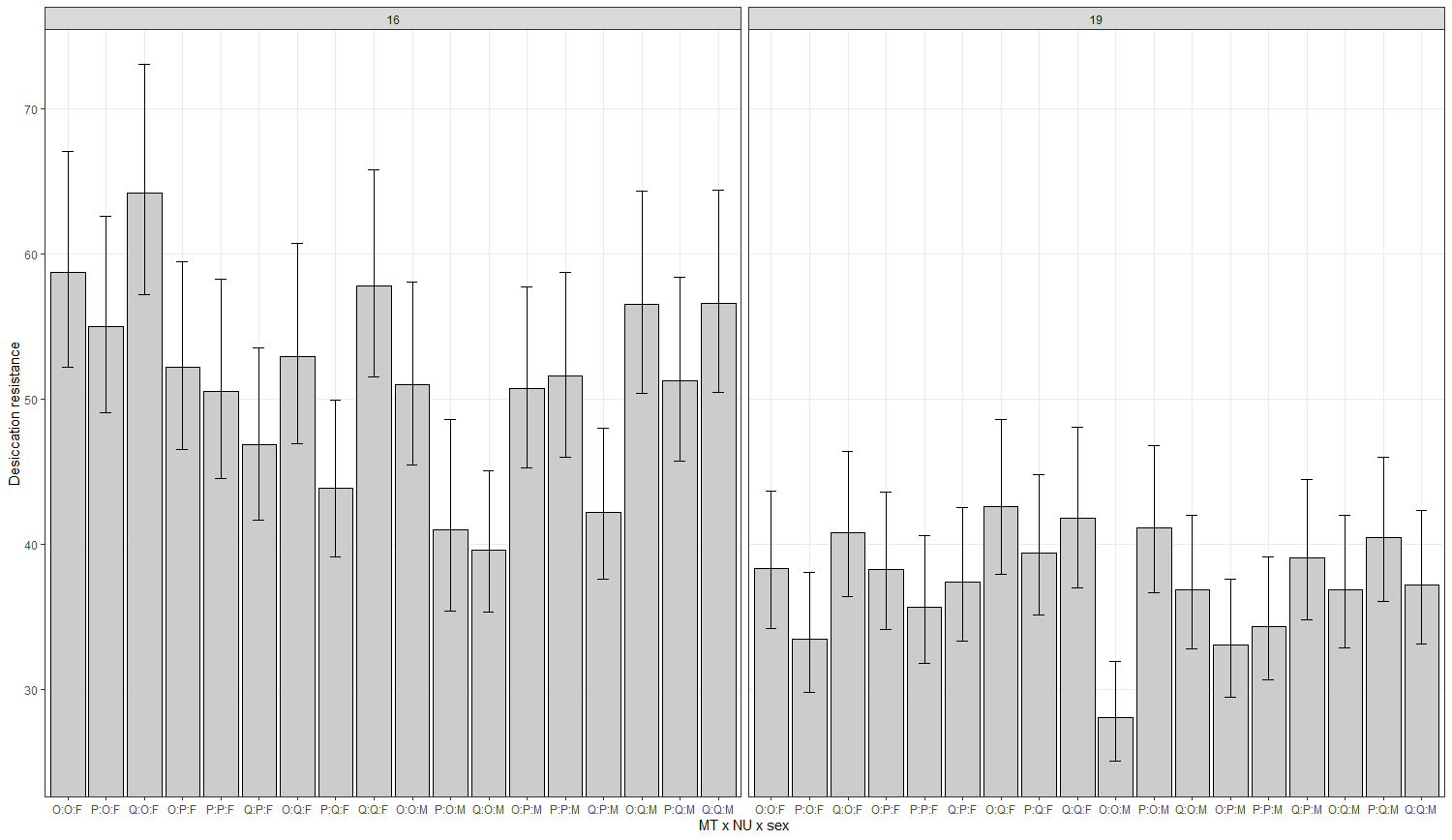

Supplement: Supplementary file 1 [file insects-13-00139-s001.zip › Supplementary Figure 4.png]

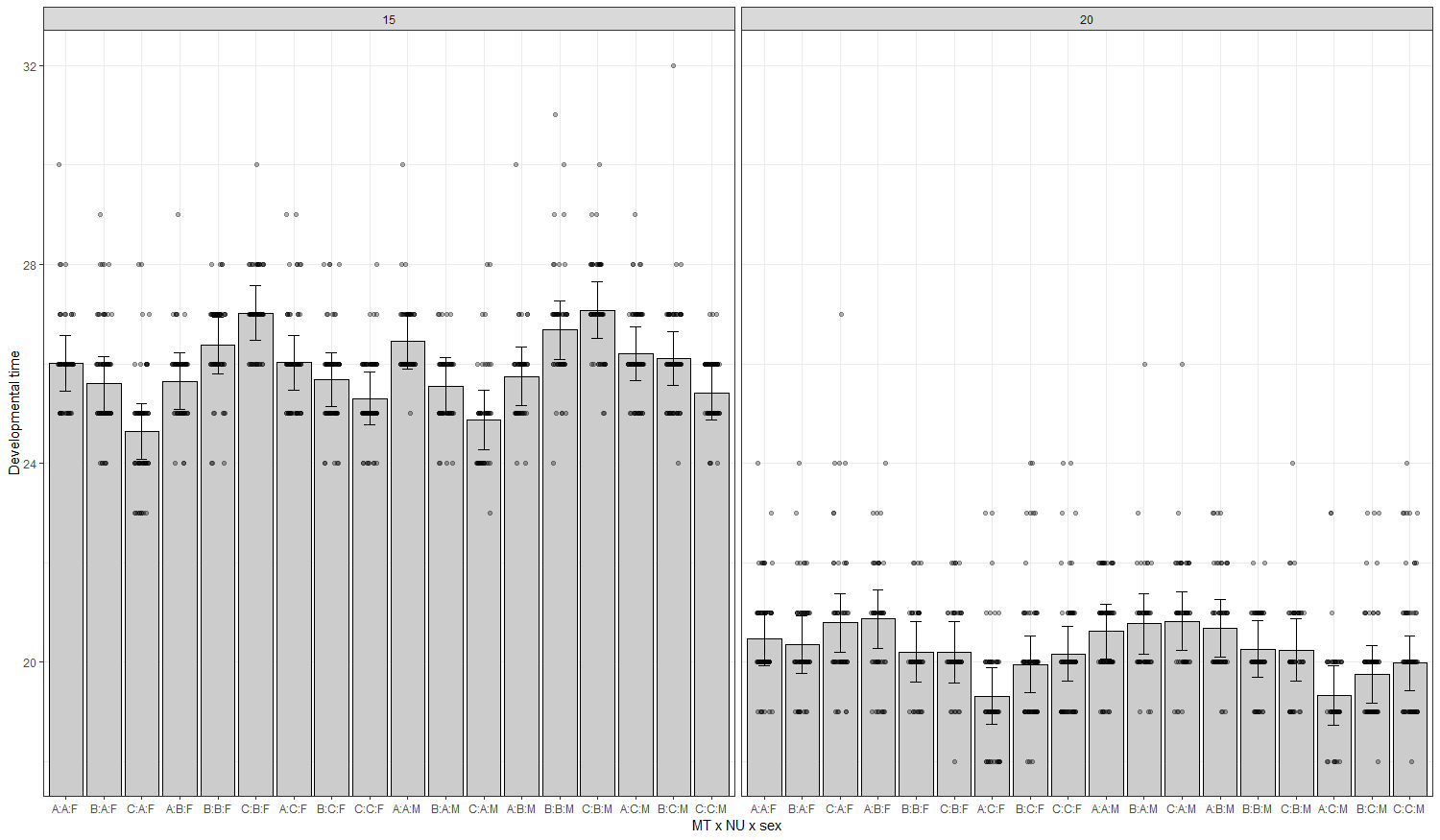

Supplement: Supplementary file 1 [file insects-13-00139-s001.zip › Supplementary Figure 5.png]

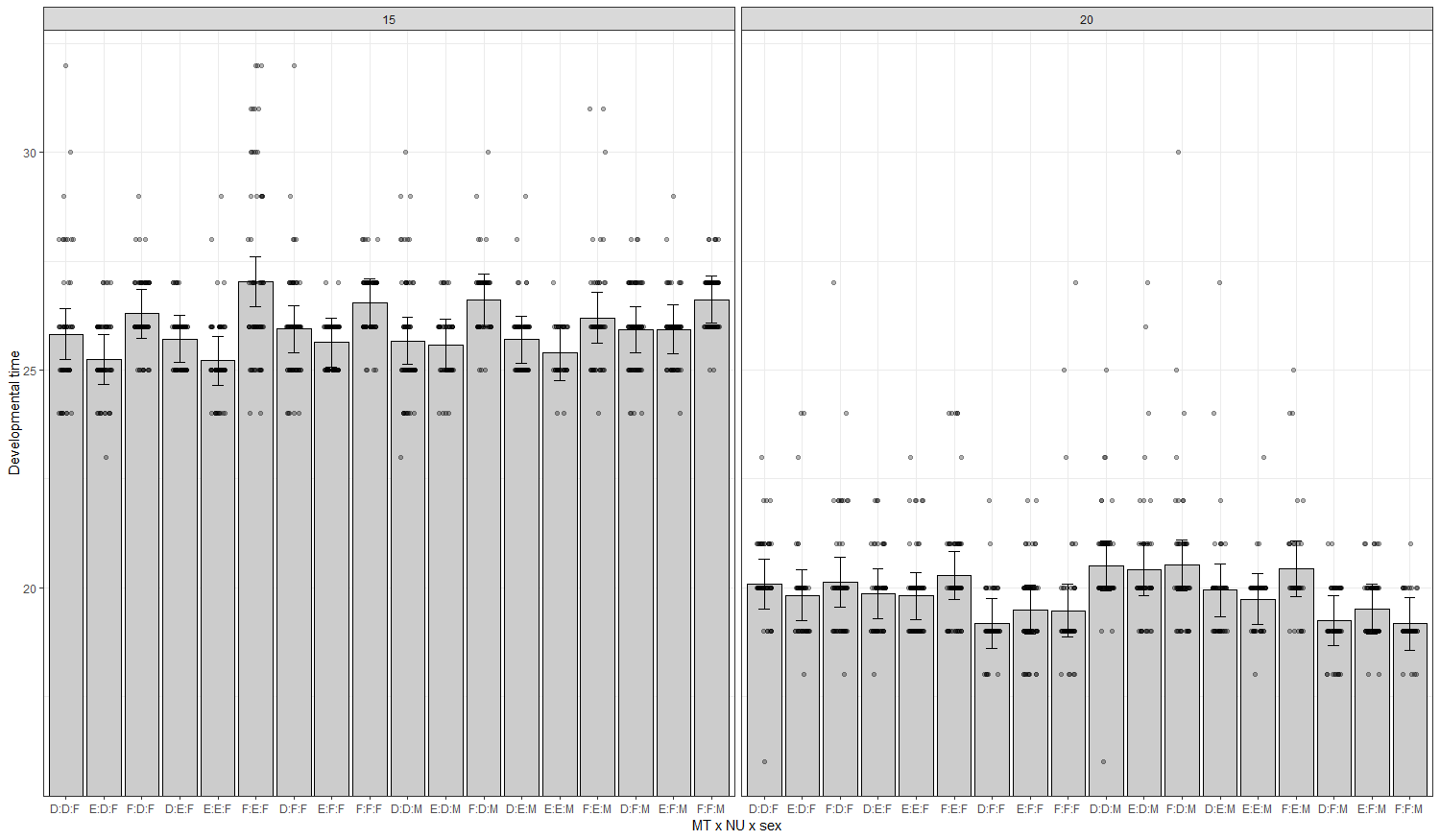

Supplement: Supplementary file 1 [file insects-13-00139-s001.zip › Supplementary Figure 6.png]

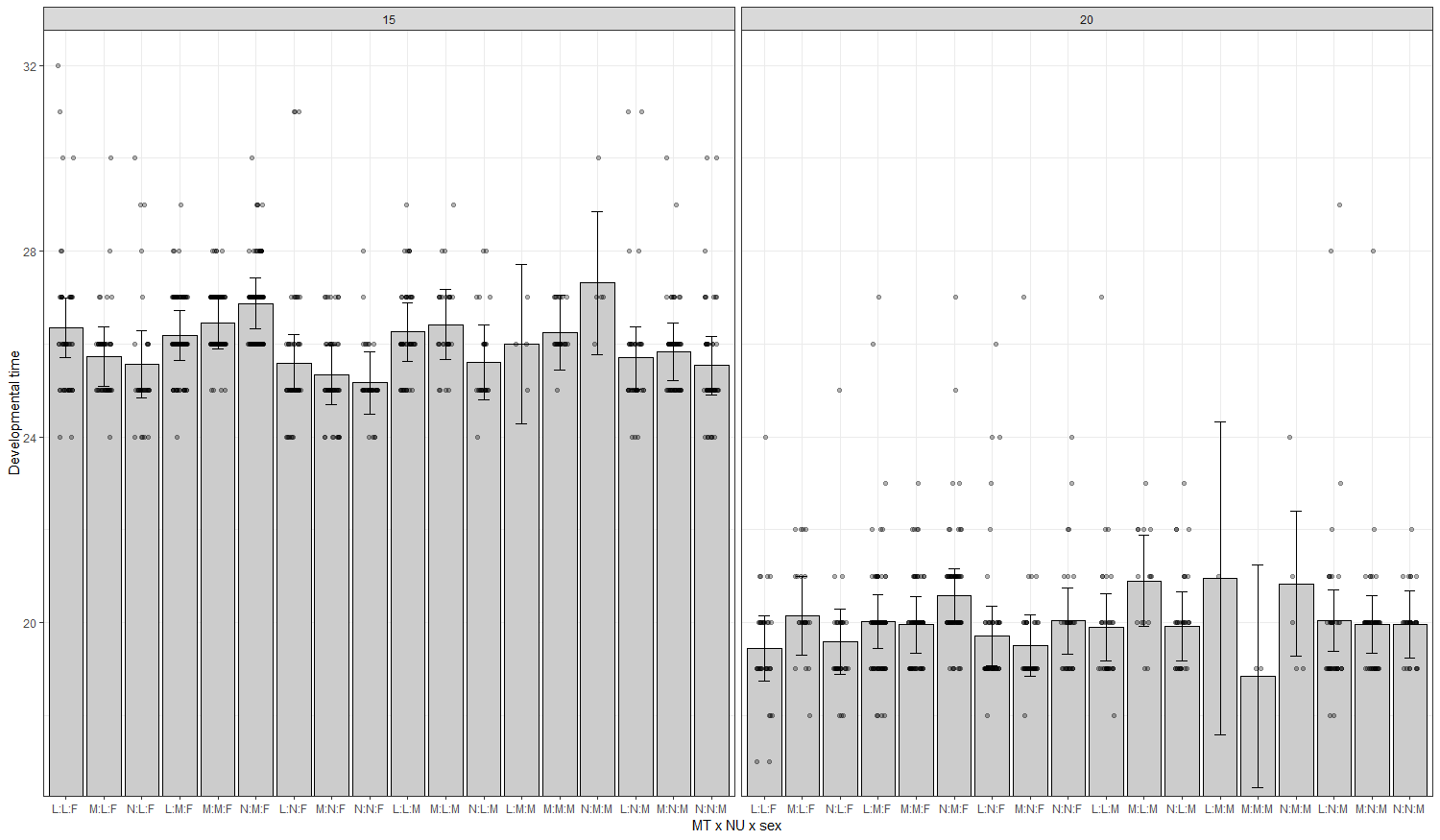

Supplement: Supplementary file 1 [file insects-13-00139-s001.zip › Supplementary Figure 7.png]

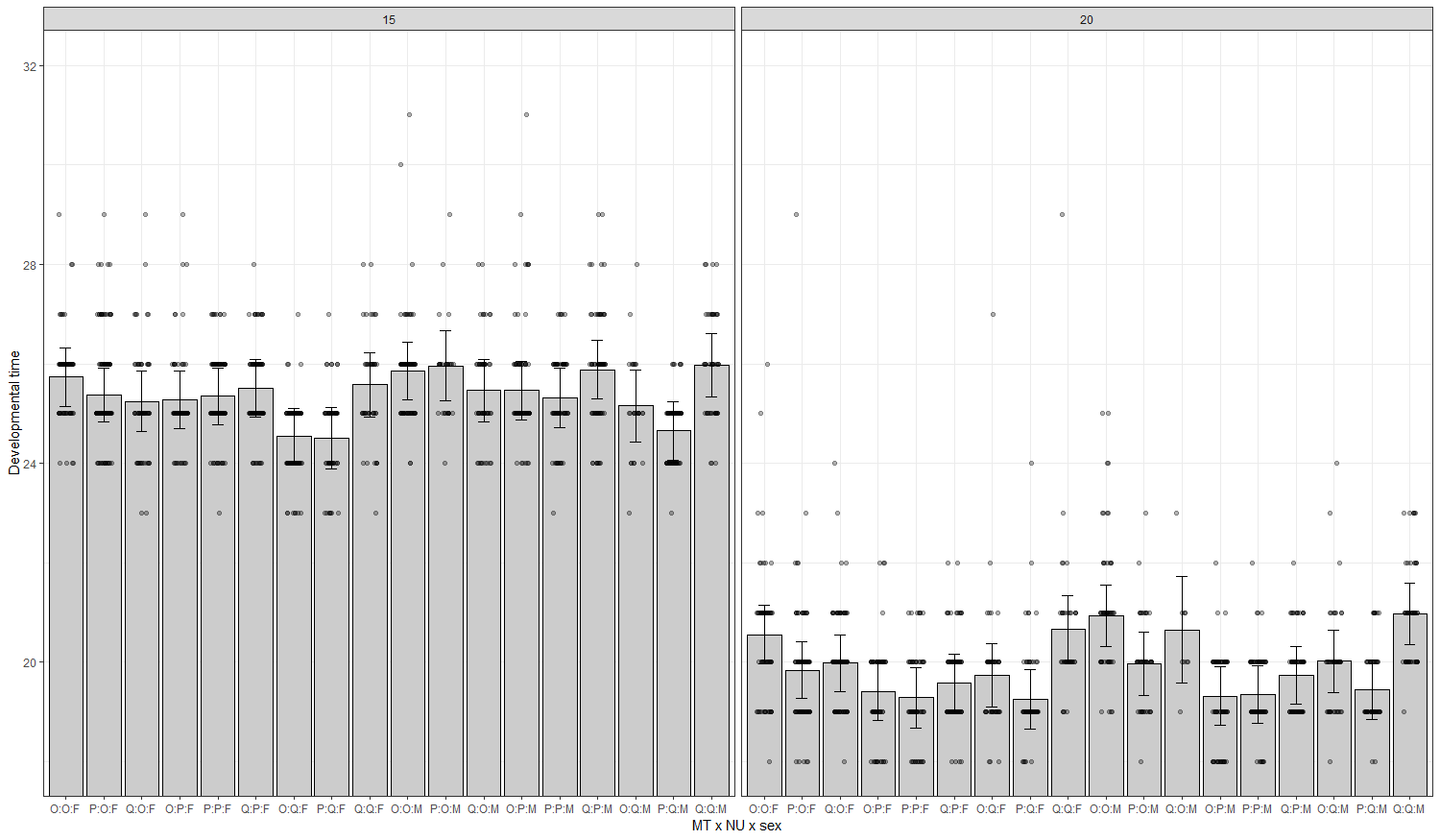

Supplement: Supplementary file 1 [file insects-13-00139-s001.zip › Supplementary Figure 8.png]

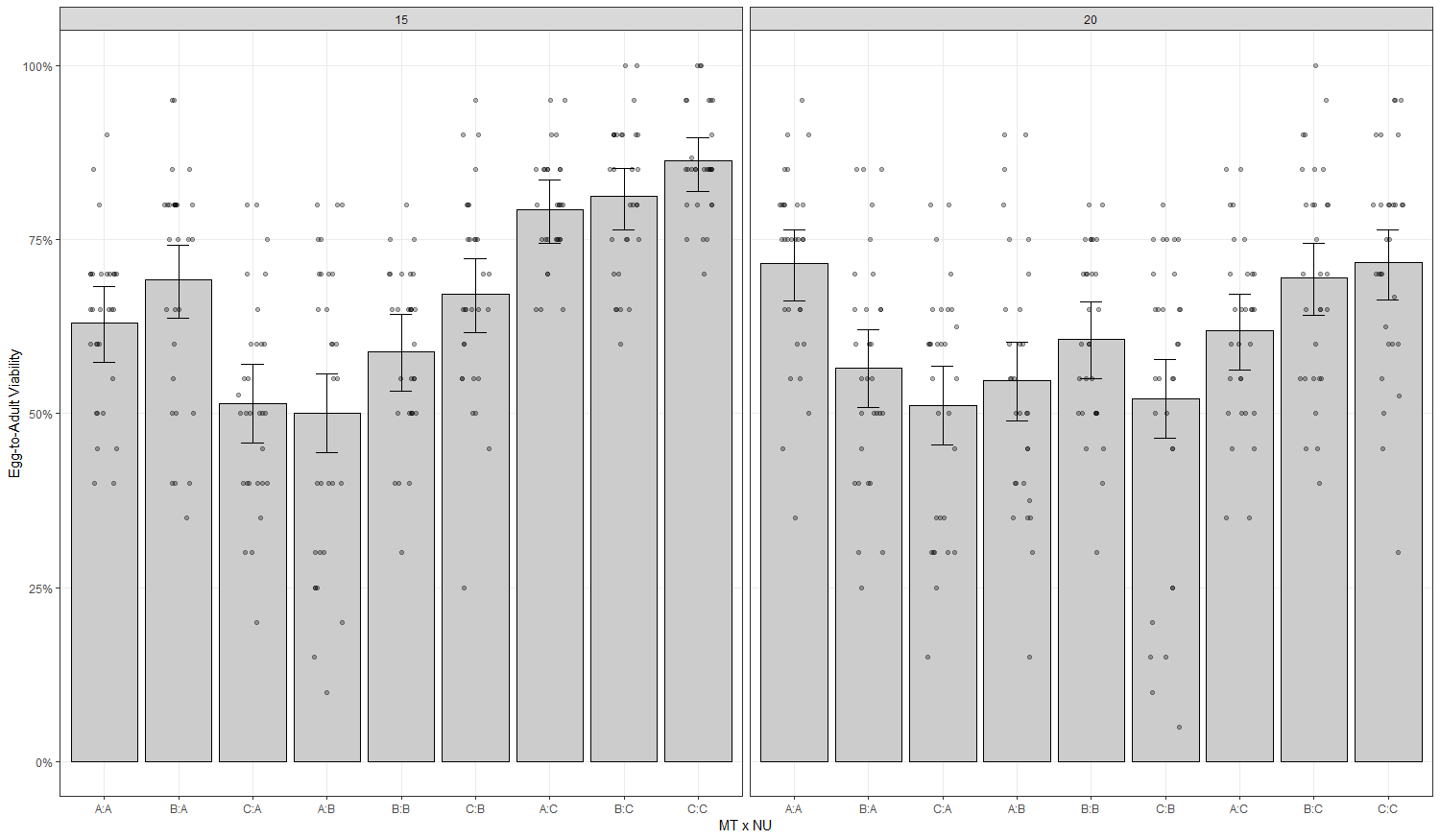

Supplement: Supplementary file 1 [file insects-13-00139-s001.zip › Supplementary Figure 9.png]
